# A Mode-Aware Hybrid Machine-Learning Framework for Full-Field Warpage Prediction of Fan-Out Panel-Level Packaging After Debonding

**DOI:** 10.3390/ma19163500

**Published:** 2026-08-18

**Authors:** Ming-Ching Huang, Yu-Ting Su, Kuo-Ning Chiang

**Affiliations:** 1 Department of Power Mechanical Engineering, National Tsing Hua University, Hsinchu 300044, Taiwan; kmc120799@gmail.com; 2 College of Semiconductor Research, National Tsing Hua University, Hsinchu 300044, Taiwan; allen16942@gmail.com

**Keywords:** Fan-Out Panel-Level Packaging, warpage prediction, hybrid machine learning, finite element method

## Abstract

Fan-Out Panel-Level Packaging (FO-PLP) enables high area utilization and manufacturing efficiency, but process-induced warpage caused by the coefficient of thermal expansion (CTE) mismatch and polymer shrinkage remains a major challenge. This study presents a classifier-gated hybrid machine-learning framework for the rapid and accurate FO-PLP warpage prediction using a database generated from a validated three-dimensional finite element process model. A Random Forest classifier first estimates the probability of each global warpage mode, while cluster analysis reduces the spatial training dataset. Two mode-specific artificial neural networks are then combined through probability-weighted fusion to predict the full warpage field and enable warpage prediction for previously unseen geometry layouts. The framework was evaluated on 16 independent finite element designs spanning both warpage modes. Compared with an equivalent single-network model, the proposed approach consistently achieved lower mean and maximum prediction errors across all designs, with the greatest improvements at the panel edges and corners where the prediction is most challenging. In addition, the clustering strategy reduced the training-set size and computational cost. These results demonstrate that integrating warpage-mode classification with mode-specific learning improves both the prediction accuracy and training efficiency, providing a practical tool for the fast warpage assessment of new FO-PLP layout designs.

## 1. Introduction

The Fan-Out Panel-Level Package (FO-PLP) discussed in this study, compared to wafer-level packaging (WLP), offers a large-area format with a high utilization efficiency and a broader distribution of I/O connections. However, due to its larger footprint, panel-level packaging is more susceptible to significant warpage during manufacturing processes [1].

FO-PLP can be implemented using the Die-first face-down, Die-first face-up, or Die-last process flows [2]. This study focuses on the Die-first face-down route, with compression molding and post-mold curing at 175 °C, followed by laser debonding at 185 °C. Although panel-level processing improves the substrate utilization and reduces the packaging cost compared with wafer-level packaging [2,3], the larger panel size amplifies the out-of-plane deformation caused by a CTE mismatch, increasing the challenges in handling and lithographic alignment. Warpage is primarily driven by EMC chemical shrinkage during curing and the CTE mismatch among the EMC, dies, and glass carrier during thermal processing [1,4]. Consequently, an accurate warpage prediction requires a rheological representation of the EMC behavior.

Thermosetting polymer materials exhibit complex nonlinear behavior during heating and cooling cycles. Therefore, in this study, a nonlinear rheological model is adopted to describe the mechanical properties and material characteristics of such materials [5,6].

The FEM is frequently used to simulate and analyze engineering problems [7,8,9,10,11,12]. However, 3D FEM simulations are often time-consuming, and the results may be affected by modeling assumptions, including the mesh strategy, interface and boundary condition definitions, and material parameter selection. To address these limitations, this study introduces a machine-learning approach to predict the post-process warpage behavior of FO-PLP. The validated FEM is employed to construct both training and testing datasets, and machine-learning techniques are then applied to analyze correlations within the data, ultimately predicting the overall warpage distribution of the package. While ensemble learning frameworks are robust for predicting the electronic packaging reliability [13,14,15,16,17,18,19,20], accurately capturing the complex warpage of FO-PLP remains a challenge. Previous studies from our laboratory have explored warpage mechanisms using equivalent CTE methods [4], but these often simplify the nonlinear material behavior during processing.

The advantages of machine learning lie in its high prediction accuracy and strong computational capability. Different engineering problems require different algorithms, and the choice of algorithm should be based on the data’s characteristics. Since panel-level packaging warpage can exhibit multiple modes and the warpage values in adjacent regions are often very close, this study designs a modeling approach tailored to these characteristics.

First, a Random Forest classifier is employed to determine the probability of each warpage mode. Next, clustering analysis, an unsupervised learning technique, is used to select representative points from the dataset as training samples, thereby reducing the computational cost of model training. Finally, an ANN is applied to predict panel-level warpage values, with warpage mode probabilities incorporated into an ensemble learning framework to enhance the prediction accuracy further. Some basic and primary concepts of this framework have been shown in our previous works [13,19,21,22,23].

## 2. Fundamental Theory

### 2.1. Nonlinear Rheological Viscoelastic–Plastic Model

In this study, the nonlinear viscoelastic–plastic rheological material model [24,25,26] is employed to simulate the warpage behavior of thermosetting polymers subjected to multiple thermal cycles. This model is also known as Burger’s rheological model.

**Figure 1:**
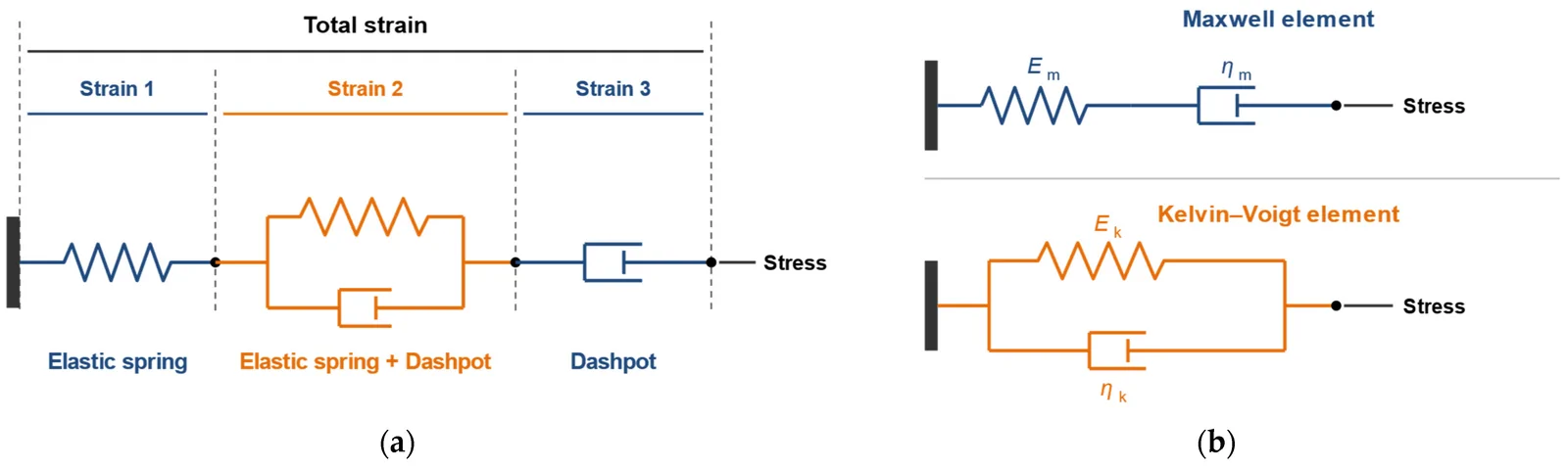
Nonlinear rheological model: (**a**) the Burgers model; and (**b**) the two elementary elements. Maxwell-element parts are drawn in blue, Kelvin–Voigt-element parts in orange.

The nonlinear rheological model is a mathematical representation composed of serially connected components, including the Maxwell model [27] and the Kelvin–Voigt model [28], as illustrated in Figure 1. Each element within the model serves a distinct function: a single spring represents the elastic behavior of the material; the parallel combination of a spring and a damper characterizes the time-dependent viscous behavior; and the individual damper describes the plastic deformation following the elastic strain.

In the Burgers model, the total strain comprises the instantaneous elastic deformation from the Maxwell spring, the delayed elastic deformation from the Kelvin–Voigt element, and the irreversible viscous deformation from the dashpot. The model parameters, including the spring constants $E_{m}$ and $E_{k}$ and the viscosities $\eta_{m}$ and $\eta_{k}$, were identified by fitting the model to EMC stress-relaxation data. Because the Burgers viscoelasticity is not directly available in ANSYS (version 2024 R2, ANSYS, Inc., Canonsburg, PA, USA), the fitted relaxation response was converted to an equivalent Prony series through the Laplace-domain transformation and term-by-term matching of the relaxation modulus [29]. The resulting Prony coefficients were then implemented in the finite element model. The Prony series formulation is the standard representation of the linear viscoelastic relaxation behavior in commercial finite element solvers [27,30].

### 2.2. Random Forest

Random Forest [31,32,33] is an algorithmic framework composed of multiple decision trees. It begins by generating multiple bootstrap subsets of the dataset, each used to train a distinct decision tree. The final prediction is obtained by aggregating the outputs of all trees: classification tasks use majority voting, while regression tasks use the average of the predictions.

### 2.3. K-Means Clustering

Cluster analysis is an unsupervised learning technique that identifies the inherent patterns and correlations within data without prior knowledge of its structure [34,35,36]. Determining the appropriate number of clusters, K, requires evaluating multiple candidate values [37,38]. The selected K should provide a sufficient representation of the dataset variability while maximizing the computational efficiency through training data reduction.

### 2.4. Artificial Neural Network

**Figure 2:**
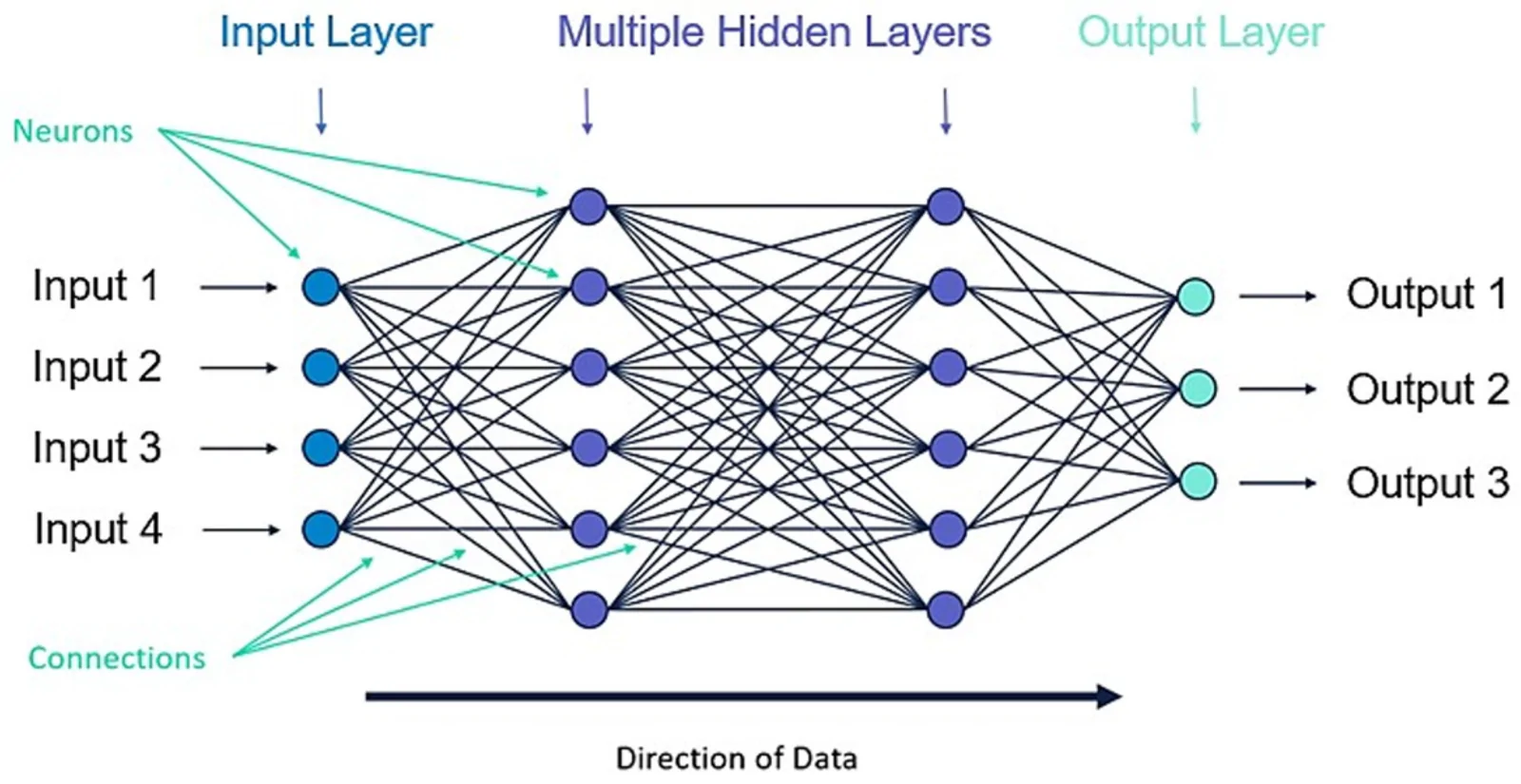
Schematic diagram of an artificial neural network.

Artificial neural networks (ANNs) are composed of interconnected neurons arranged in the input, hidden, and output layers, as illustrated in Figure 2 [39,40,41]. During training, connection weights are iteratively updated through backpropagation to minimize a loss function representing the prediction error. The predictive performance of an ANN depends strongly on the selection and optimization of key hyperparameters.

## 3. Finite Element Analysis of FO-PLP

In this study, a Fan-Out Panel-Level Packaging (FO-PLP) model was developed using the FEM in ANSYS^®^. By integrating process modeling technology, the simulation better reflects actual manufacturing conditions.

### 3.1. Finite Element Model

The material parameters considered in model construction include the CTE, Young’s modulus, Poisson’s ratio, and material density. Among these, the mismatch in CTE between different materials is the primary cause of package warpage, making CTE one of the most critical parameters. The material properties are summarized in Table 1 [22]. In addition, the CTE of the EMC exhibits temperature-dependent behavior, as shown in Table 2 [22].

Due to the complex and variable structure of the EMC, a nonlinear rheological model is adopted in this study to describe its influence on the overall warpage during multiple thermal cycles in the manufacturing process. The parameters of the nonlinear rheological model components are listed in Table 3. These component parameters are then converted into a Prony series for finite element analysis, as shown in Table 4.

The finite element settings are summarized in Table 5; the element types and mesh density follow the mesh size control study previously reported for FO-PLP panel models [4].

**Figure 3:**
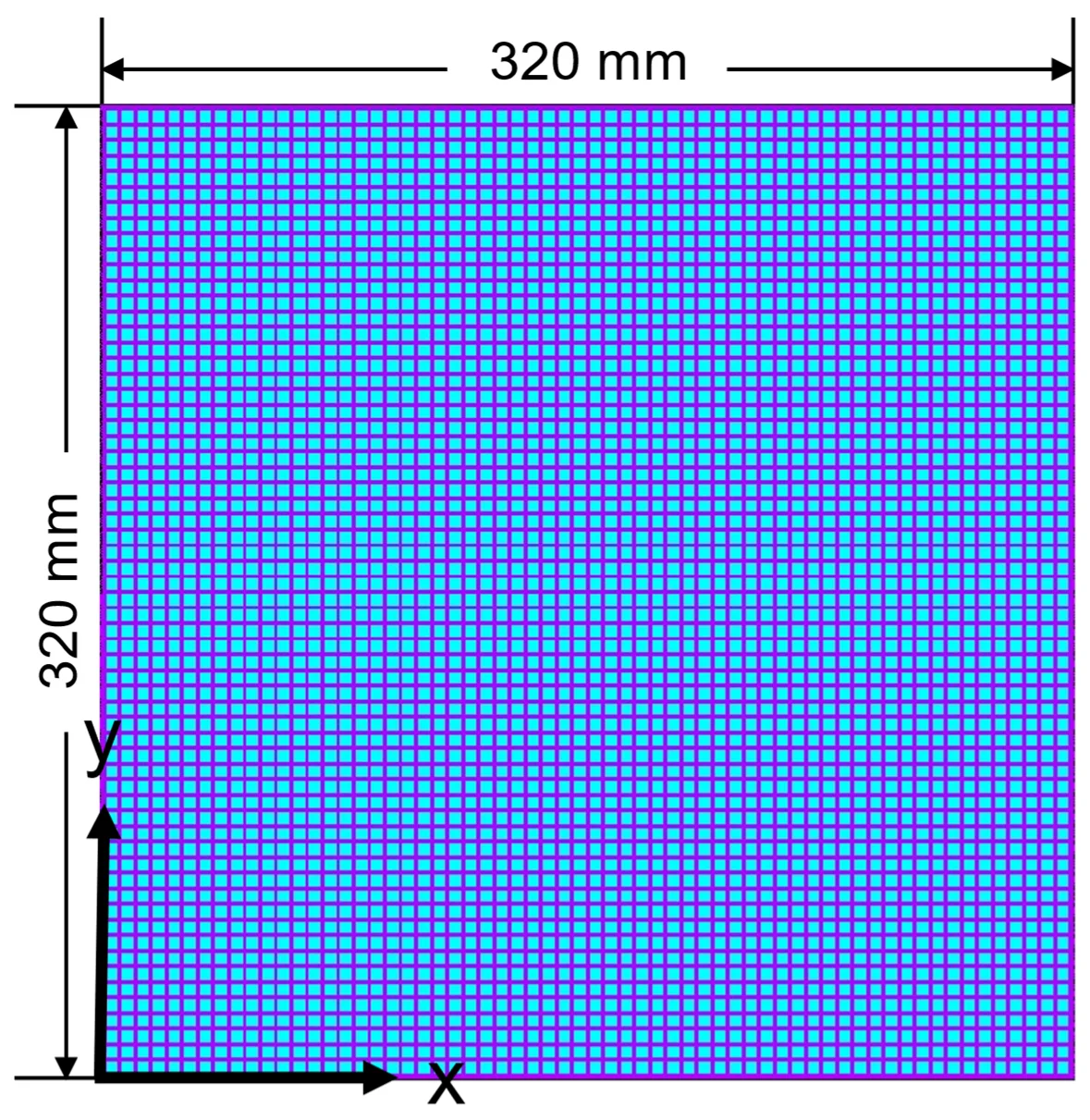
Top view of the FO-PLP model [23].

**Figure 4:**
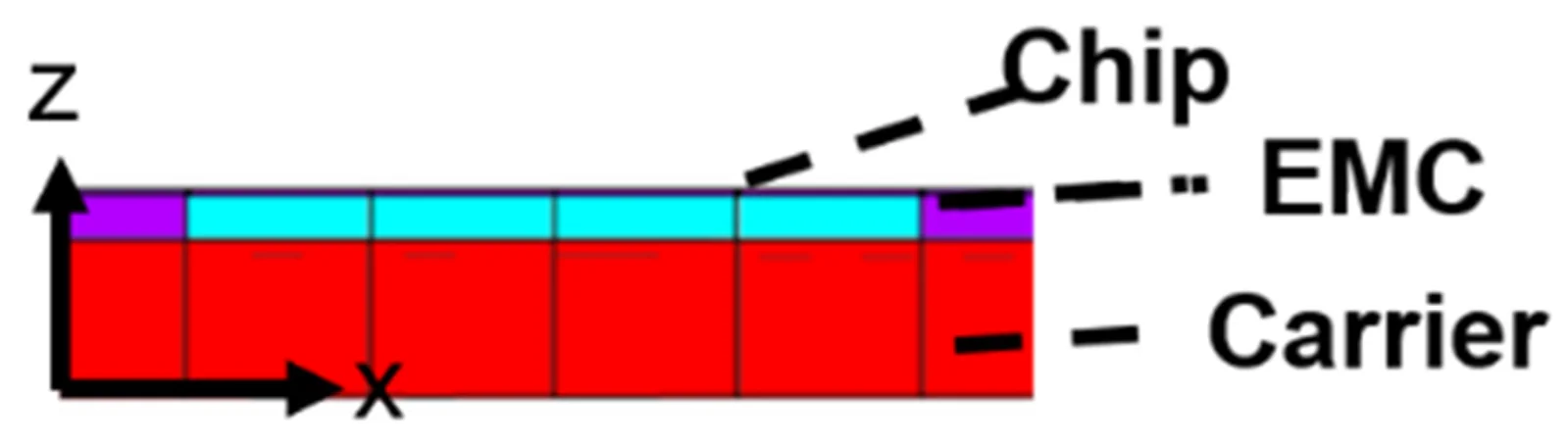
Side view of the FO-PLP model [23].

In this study, the Fan-Out Panel-Level Packaging (FO-PLP) structure with dimensions of 320 mm × 320 mm, as proposed by Su et al. [7], is adopted as the basis for model development. A full-chip is designed in both the x and y directions. Additionally, the feature sizes of each material layer, such as chip thickness, chip size, chip spacing, EMC thickness, and carrier thickness, are defined based on our previous study to construct the finite element model, as illustrated in Figure 3 and Figure 4 [23].

The viscoplastic Burgers model and the process-modeling procedure adopted here have been validated against experimental measurements in our previous work, reproducing measured warpage to within 4.5% [22] and 0.28–0.52% [4].

### 3.2. Boundary Condition and Loading Setting

The constructed model represents an FO-PLP. To prevent rigid-body motion, all degrees of freedom are constrained at the panel’s central node. Gravitational effects are also considered during the process simulation, and, thus, gravity is applied to the model.

After the Compression Molding and PMC processes, the simulation proceeds to the debonding stage. In practical manufacturing, laser debonding is conducted in a vacuum chamber, with the parts released instantaneously. To replicate this behavior, the present study simulates the vacuum adsorption and release mechanisms by incorporating multiple spring elements.

**Figure 5:**
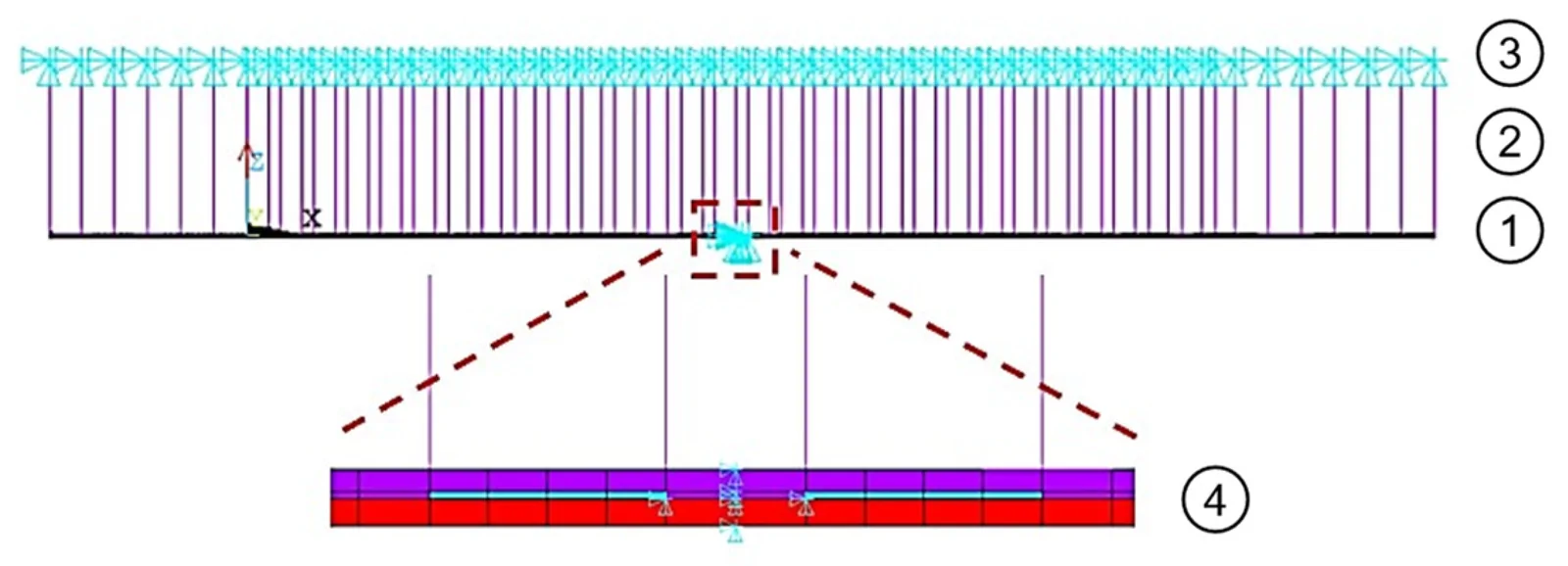
Illustration of the connection between spring elements and the FO-PLP model: ① the upper surface of the EMC; ② the spring elements; ③ the vacuum chuck; and ④ the panel cross-section.

These springs are created by connecting the upper nodes of the EMC layer to the top nodes of the spring elements. The spring element type used is COMBIN14, which is massless and, therefore, does not require gravity consideration. The configuration of the spring connections is illustrated in Figure 5.

Before the simulation begins, the spring elements are deactivated. During debonding, these elements are activated to emulate the release behavior.

The process simulation procedure is summarized in Table 6. Initially, the entire model is constructed, including the glass carrier, chips, EMC, and spring elements. In the early stages of the simulation, only the glass carrier and chips are active, while the EMC and spring elements are deactivated using element death techniques.

The simulation begins by applying a temperature load that ramps from room temperature to the molding temperature. Once the molding temperature is reached, the EMC elements are reactivated to simulate the molding process. The temperature is held at the molding temperature for a specified time, then cooled back to room temperature.

The simulation then proceeds to the PMC stage, where the temperature is held constant for a period. After another cooling phase to room temperature, the debonding process begins.

At the start of the debonding stage, the spring elements are reactivated to simulate vacuum adsorption. The spring stiffness is set to a very high value to ensure strong adhesion. During heating to the debonding temperature, care is taken to prevent excessive deformation that may lead to convergence issues in the finite element simulation.

In the vacuum release phase, the carrier is gradually peeled off from both sides to avoid sudden warpage changes that could cause numerical instability. After the debonding is completed, the model is cooled back to room temperature. The spring stiffness is then gradually reduced, and, once sufficiently small, all spring elements are deactivated to simulate the vacuum release behavior.

## 4. Hybrid Machine-Learning Framework

In this study, a finite element model of FO-PLP was developed, and a comprehensive database was successfully constructed for various feature sizes. A hybrid machine-learning framework is proposed to predict the warpage behavior based on this dataset.

The method begins by using a Random Forest to estimate the probability of each possible warpage mode (e.g., saddle-shaped, and bowl-shaped), enabling the classification of the warpage type. These probabilities are then used as weights to incorporate the likelihood of each mode, thereby improving the subsequent ANN model’s prediction accuracy.

To reduce the data volume and training time, clustering analysis is applied to the spatial warpage field to select representative locations (spatial points) on the panel, which are then used as training data for the ANN. This approach effectively captures critical information while improving the computational efficiency.

**Figure 6:**
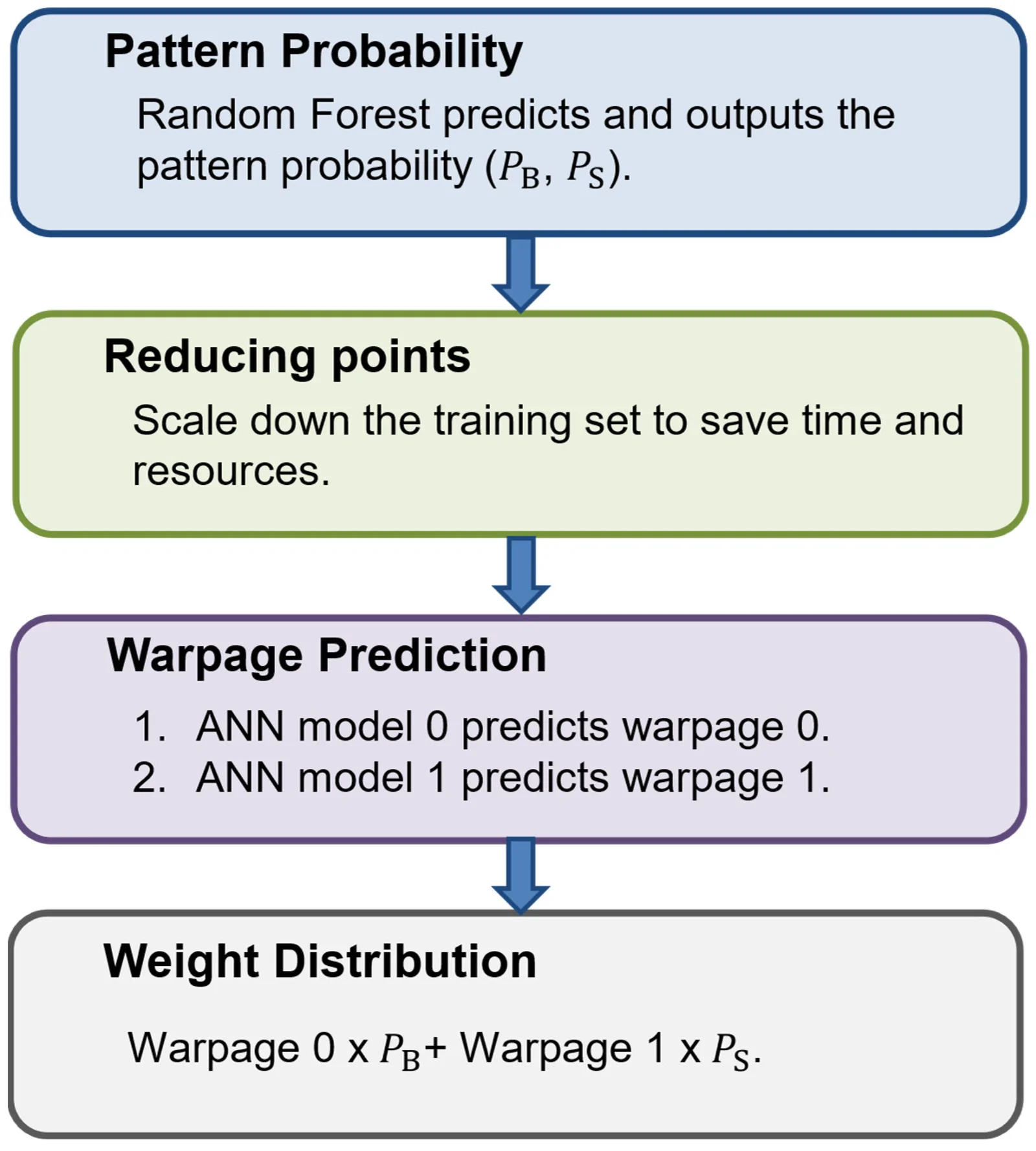
Framework of the hybrid machine-learning method.

By integrating multiple machine-learning techniques, the proposed method leverages the strengths of each to enhance the prediction accuracy and reduce the training time. The overall framework of the proposed model is illustrated in Figure 6.

### 4.1. Database

**Figure 7:**
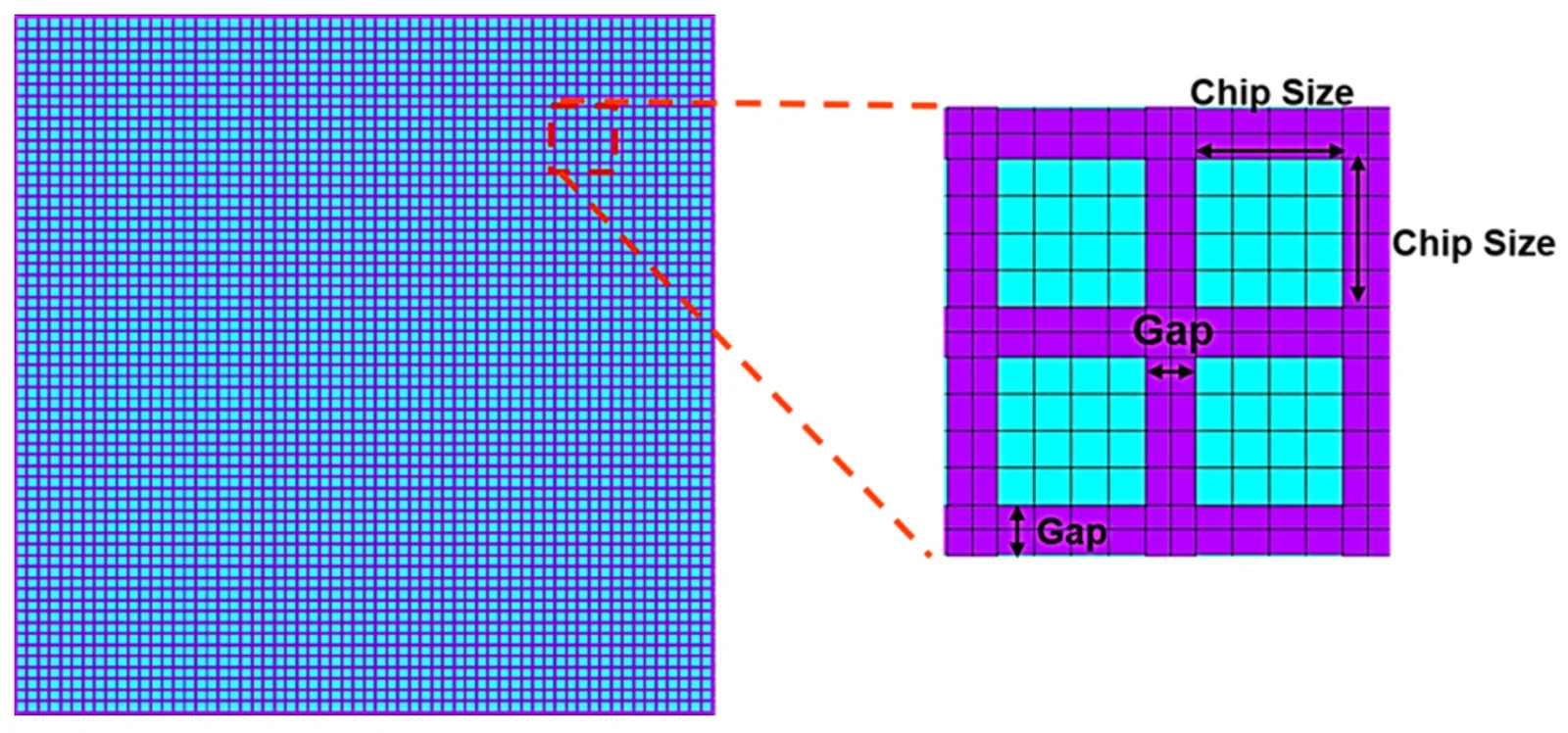
Top view of the geometric dimensions [23].

**Figure 8:**
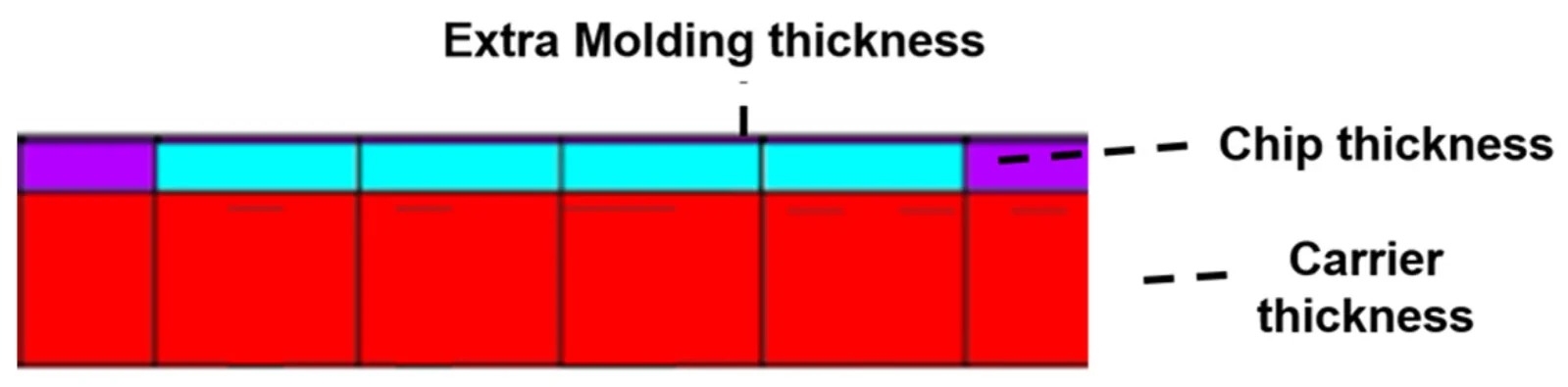
Side view of the geometric dimensions [23].

Several key feature sizes in FO-PLP are closely related to warpage behavior. In this study, five geometric parameters of the package structure are selected: the chip thickness, chip size, chip-to-chip gap, EMC thickness above the chips, and glass carrier thickness, as summarized in Table 7. The geometric features are illustrated in the top and side views of the package shown in Figure 7 and Figure 8, respectively [23].

**Figure 9:**
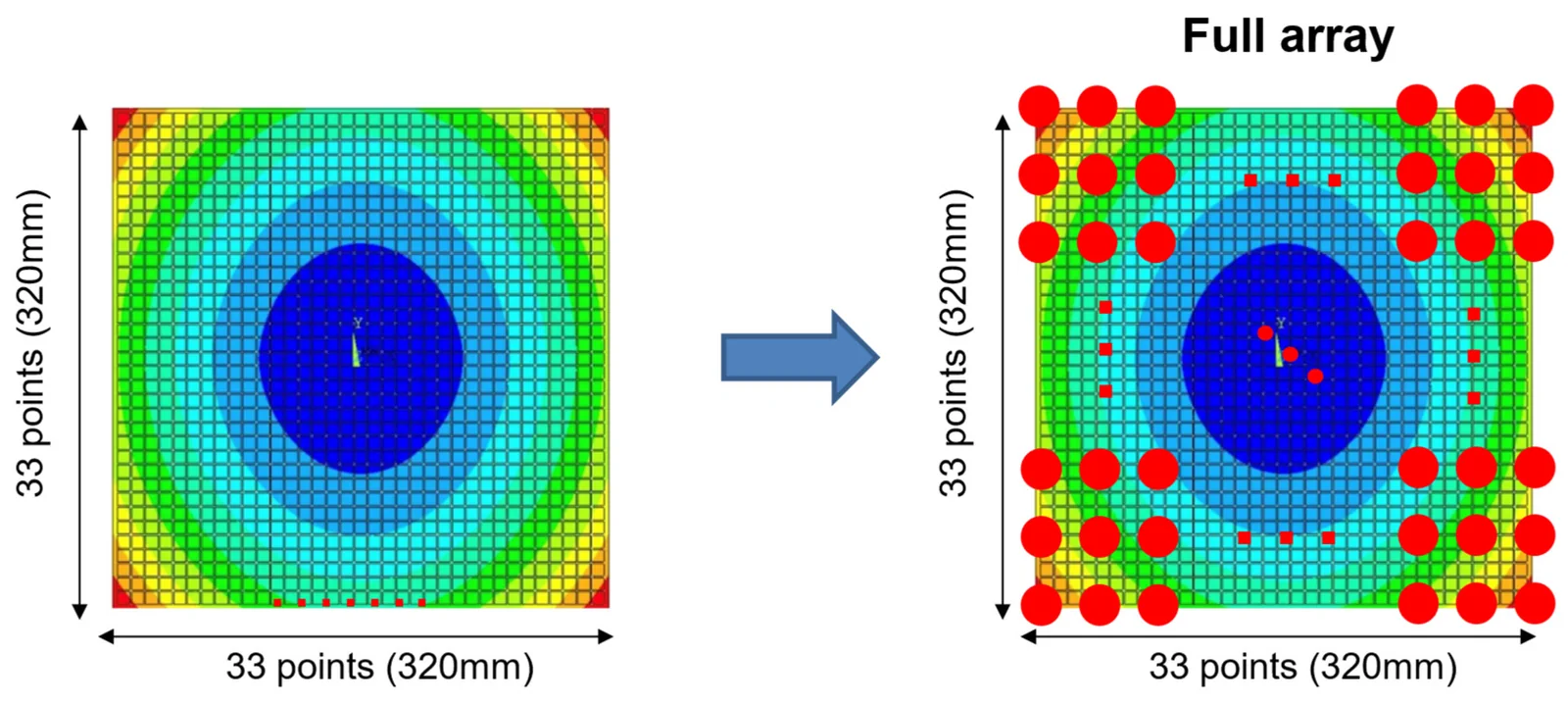
Full array method [23]. Red markers indicate the 33 × 33 sampling points, spaced 10 mm apart.

After defining the five geometric features, a dataset comprising multiple design samples (each representing a unique combination of geometric parameters) was constructed. For each design sample, spatial warpage data points were extracted using the Full Array Method. The 320 mm × 320 mm panel was divided at 10 mm intervals in both the x and y directions, resulting in 1089 spatial points per design sample, as illustrated in Figure 9 [23]. Each point corresponds to specific X and Y coordinates on the package; the Z coordinate represents the associated warpage value. The training and testing datasets are summarized in Table 8 and Table 9, respectively. Since each design sample contains 1089 spatial points, the training dataset contains 264,627 pointwise warpage entries. Two further designs, Test Sample 1 and Test Sample 2, were used during development to determine the number of clusters and to optimize the model hyperparameters, and are reported separately in Section 5.1 and Section 5.2 as illustrative cases. Sixteen additional designs, disjoint from both the training grid and the two development samples, were held out from every stage of the model selection and are used for all subsequent evaluations.

### 4.2. Warpage Mode

Compared to wafer-level packaging, panel-level packaging offers the advantage of a higher utilization efficiency. However, it is often accompanied by severe warpage during manufacturing. Depending on the degree of warpage and the influence of various geometric features, panel-level packages may exhibit different warpage modes. In the absence of geometric and material uncertainties [42], the warpage behavior tends to follow uniform deformation modes.

**Figure 10:**
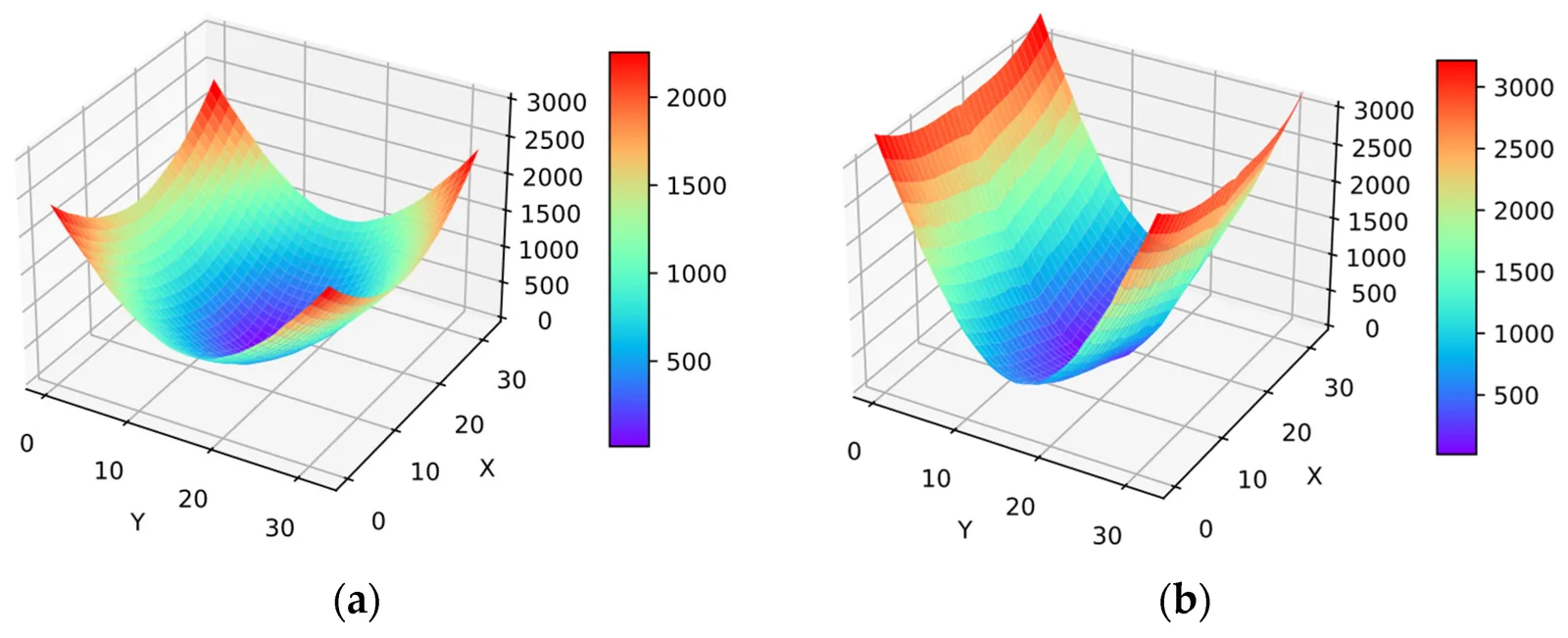
The two primary warpage modes: (**a**) bowl-shaped; and (**b**) saddle-shaped [21].

The database constructed in this study includes two primary warpage modes: bowl-shaped and saddle-shaped, as illustrated in Figure 10 [21]. Bowl- and saddle-type warpage are the two predominant deformation modes reported for panel-level packages in the literature [8,9].

Bowl-shaped warpage refers to a deformation mode in which all four corners deform in the same direction relative to the center, resulting in a symmetric concave or convex shape. This type accounts for approximately 37% of the dataset.

Saddle-shaped warpage refers to a deformation mode in which opposite corners deform in opposite directions relative to the center. This type accounts for approximately 63% of the dataset. In this study, the warpage mode label was determined based on the sign distribution of the out-of-plane displacement at the four panel corners.

Due to the variations in warpage modes, warpage values along the four edges of the panel can fluctuate significantly, severely impacting the prediction accuracy at the periphery of panel-level packages. To address this issue, this study employs a Random Forest to identify the probability of each warpage mode. These probabilities are then used as effective weights for the ANN, thereby enhancing the overall prediction accuracy.

### 4.3. Point Selection

**Figure 11:**
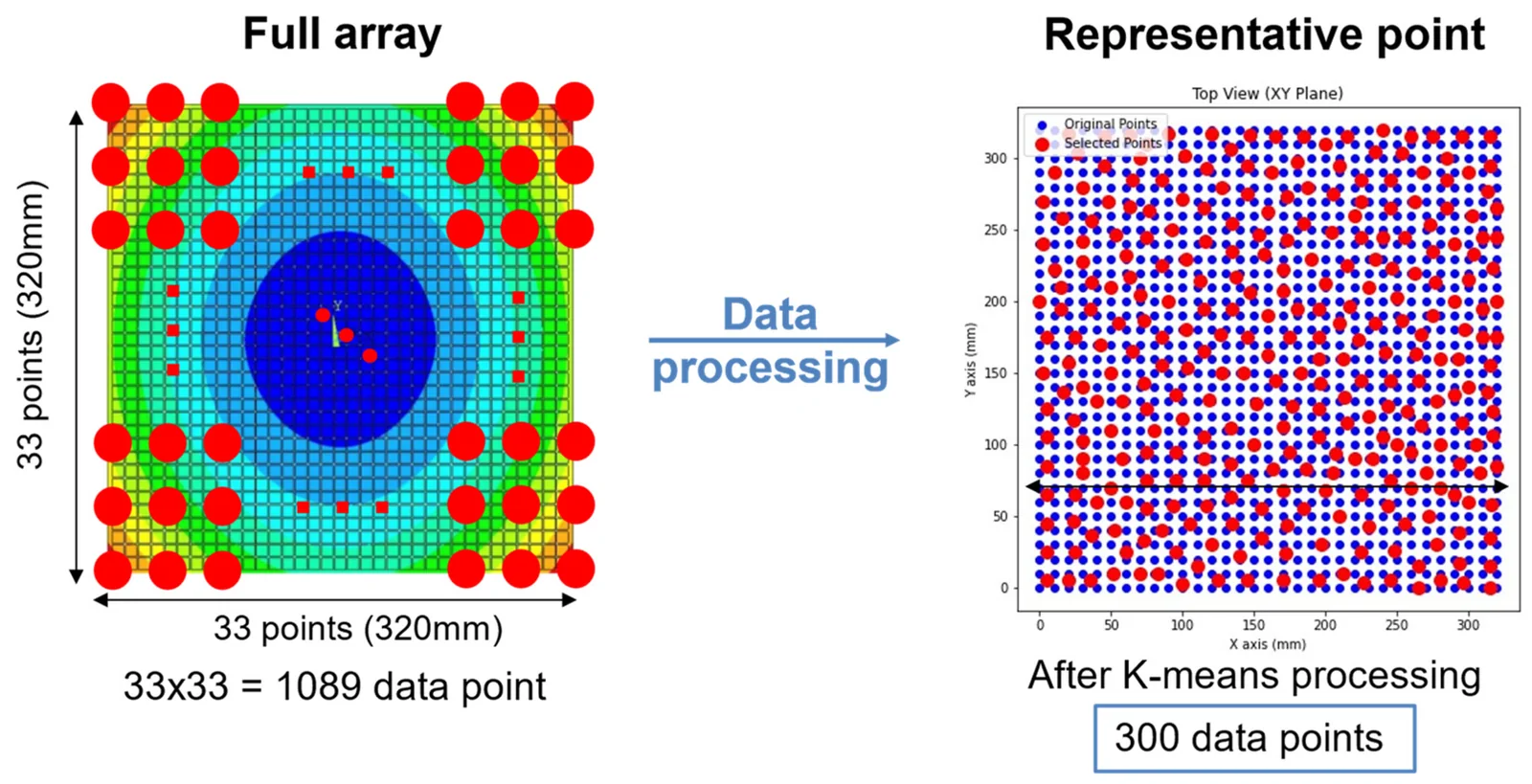
Schematic illustration of clustering results [23]. Red markers indicate the points retained after K-means reduction.

Cluster analysis is a data reduction technique that groups data based on shared characteristics. Within each cluster, the data points exhibit similar features. By calculating either the average of the points within a cluster or the distances among them, a representative point can be identified. These representative points capture the underlying characteristics of their respective clusters and, when considered collectively, can reflect the overall warpage behavior of the FO-PLP. This approach reduces the number of training data points required and effectively lowers the computational cost of model training. A schematic of the representative point selection is shown in Figure 11 [23].

Since clustering is an unsupervised learning method, there is no direct ground-truth label for evaluating the clustering accuracy. Therefore, the selection of *K* is mainly determined by the stability of the prediction performance after integrating clustering with ANN training.

## 5. Results and Discussion

### 5.1. Single ANN Model

**Figure 12:**
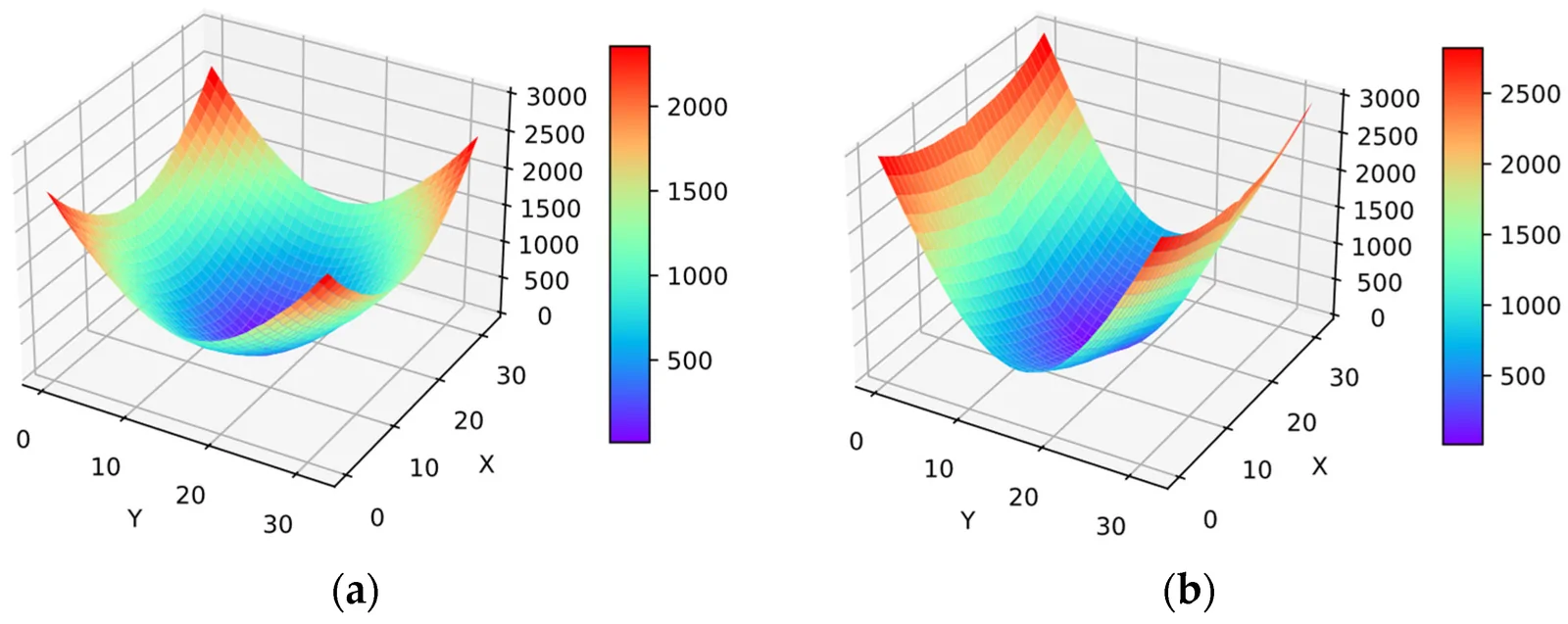
The two test designs used for the detailed illustration: (**a**) Test Sample 1, bowl-dominant; and (**b**) Test Sample 2, saddle-dominant.

This study first investigates the performance of a single ANN in warpage prediction. To optimize the network architecture, a comprehensive grid search was conducted, with the hyperparameter search space established based on our prior methodology [23]. The hyperparameter search space is summarized in Table 10, and the selected optimal combination is listed in Table 11. Two sets of test samples were used for the evaluation, as illustrated in Figure 12.

**Figure 13:**
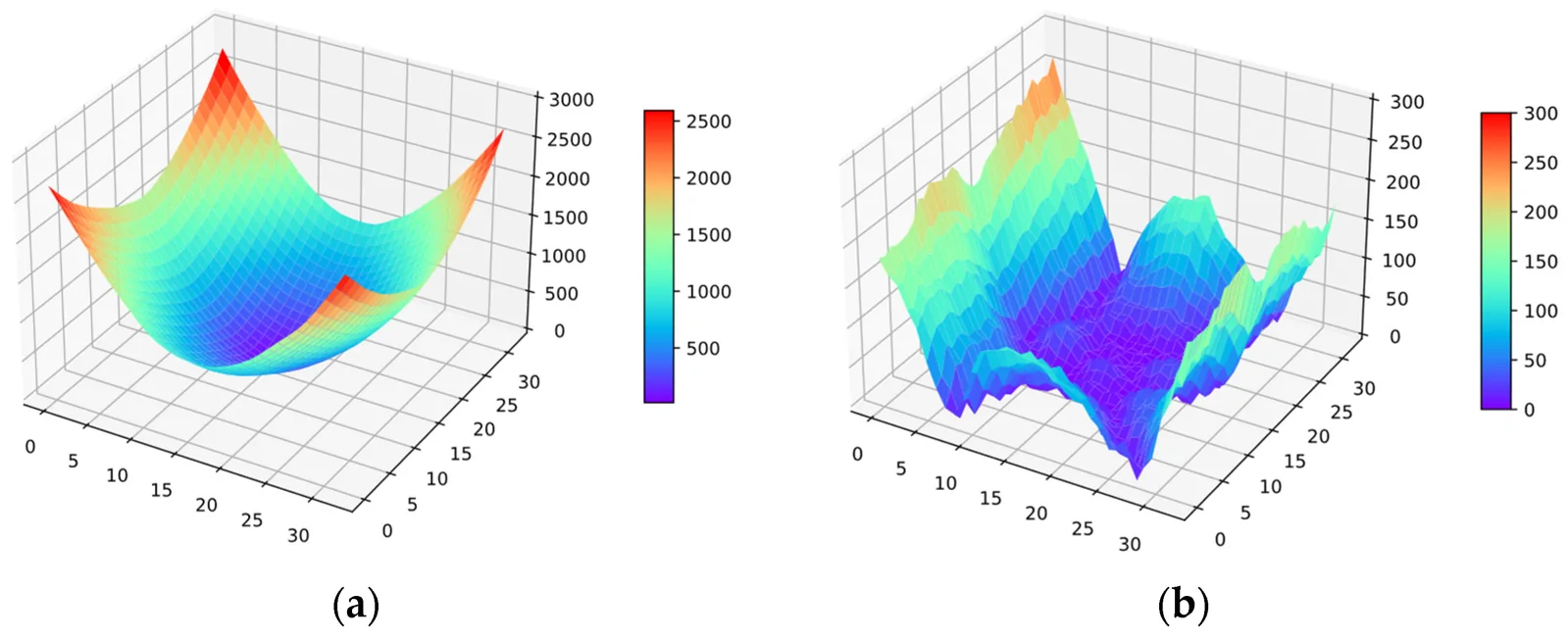
Test Sample 1 predicted by the ungated baseline: (**a**) predicted warpage field; and (**b**) absolute error surface. Axes in mm, warpage and error in µm.

The prediction was first performed on Test Sample 1, with the results presented in Table 12 and Figure 13. MAE and MAPE denote the mean absolute and mean absolute percentage error, each averaged over the N = 1089 spatial points of a design; the maximum error and its percentage refer to the single worst point. While the mean testing error was relatively low, the maximum testing error reached 10.69%, corresponding to 265.07 µm. The error surface in Figure 13b shows that the largest deviations occur at the outer regions of the package.

**Figure 14:**
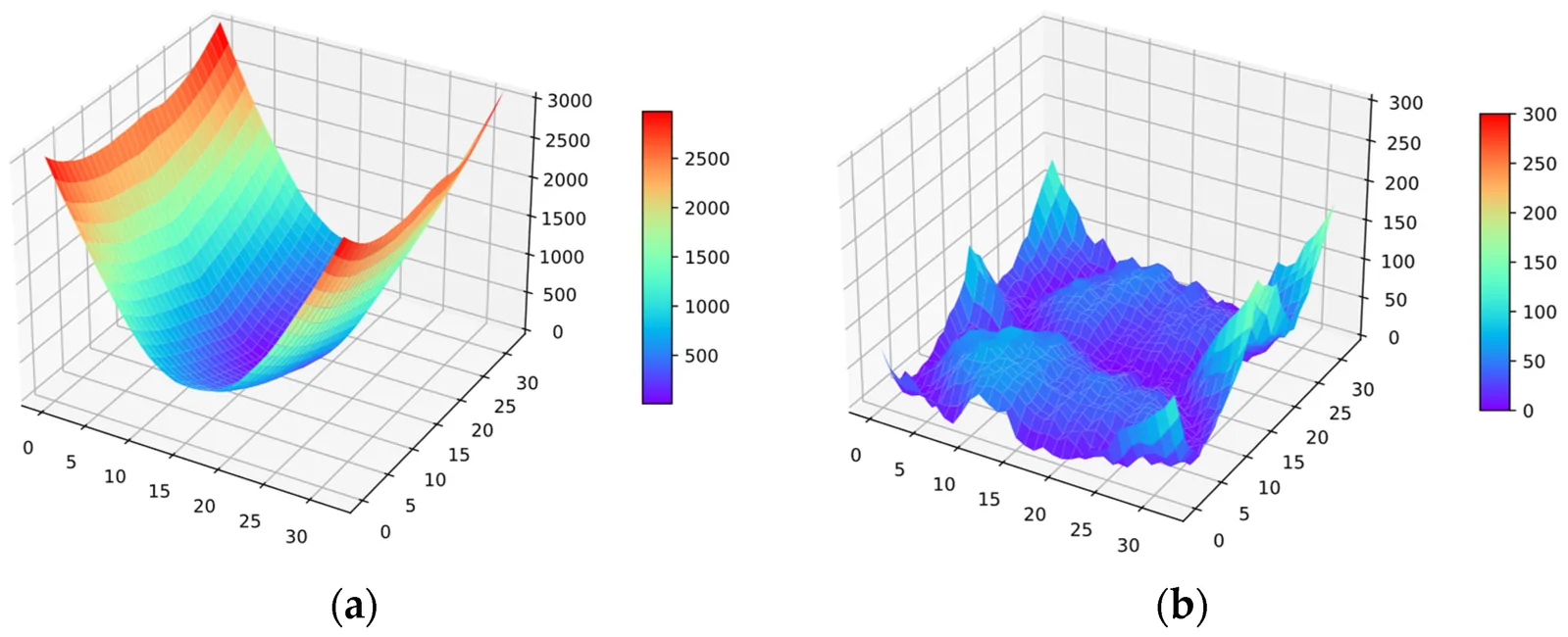
Test Sample 2 predicted by the ungated baseline: (**a**) predicted warpage field; and (**b**) absolute error surface. Axes in mm, warpage and error in µm.

Subsequently, warpage prediction was performed for Test Sample 2. The prediction results for Test Sample 2 are given in Table 12 and Figure 14, in which Figure 14a shows the predicted warpage field and Figure 14b the corresponding error surface [21]. As in the previous case, larger prediction errors are observed in the outer regions of the package.

The overall prediction results, along with observations from the training dataset, indicate that using a single ANN for warpage prediction may lead to significant errors, particularly at the outer regions of the FO-PLP. This is primarily because the relationship between geometric parameters and the spatial warpage field differs significantly between the bowl-shaped and saddle-shaped deformation modes. As a result, a single ANN trained on mixed modes tends to average these behaviors, leading to larger prediction deviations, particularly near the panel edges.

To address this issue, this study proposes a warpage prediction approach based on Ensemble Learning. The predicted probabilities of different possible warpage modes, obtained using a Random Forest, are used as weights to integrate the outputs of multiple ANN models, thereby improving the overall prediction accuracy.

### 5.2. Hybrid Machine Learning

To address the large prediction errors observed in the outer regions of the package, this study introduces a hybrid warpage prediction approach. Prior to predicting the warpage magnitude, a Random Forest is employed to estimate the probability of each warpage mode for a given sample. These probabilities are then used as weights in the subsequent prediction stage to guide the ANN models.

Two separate ANN models are trained using datasets corresponding to different warpage modes. Before ANN training, cluster analysis is applied to reduce the number of data points in the training set, thereby simplifying the dataset and improving the computational efficiency. Each sample is then predicted using both ANN models.

**Figure 15:**
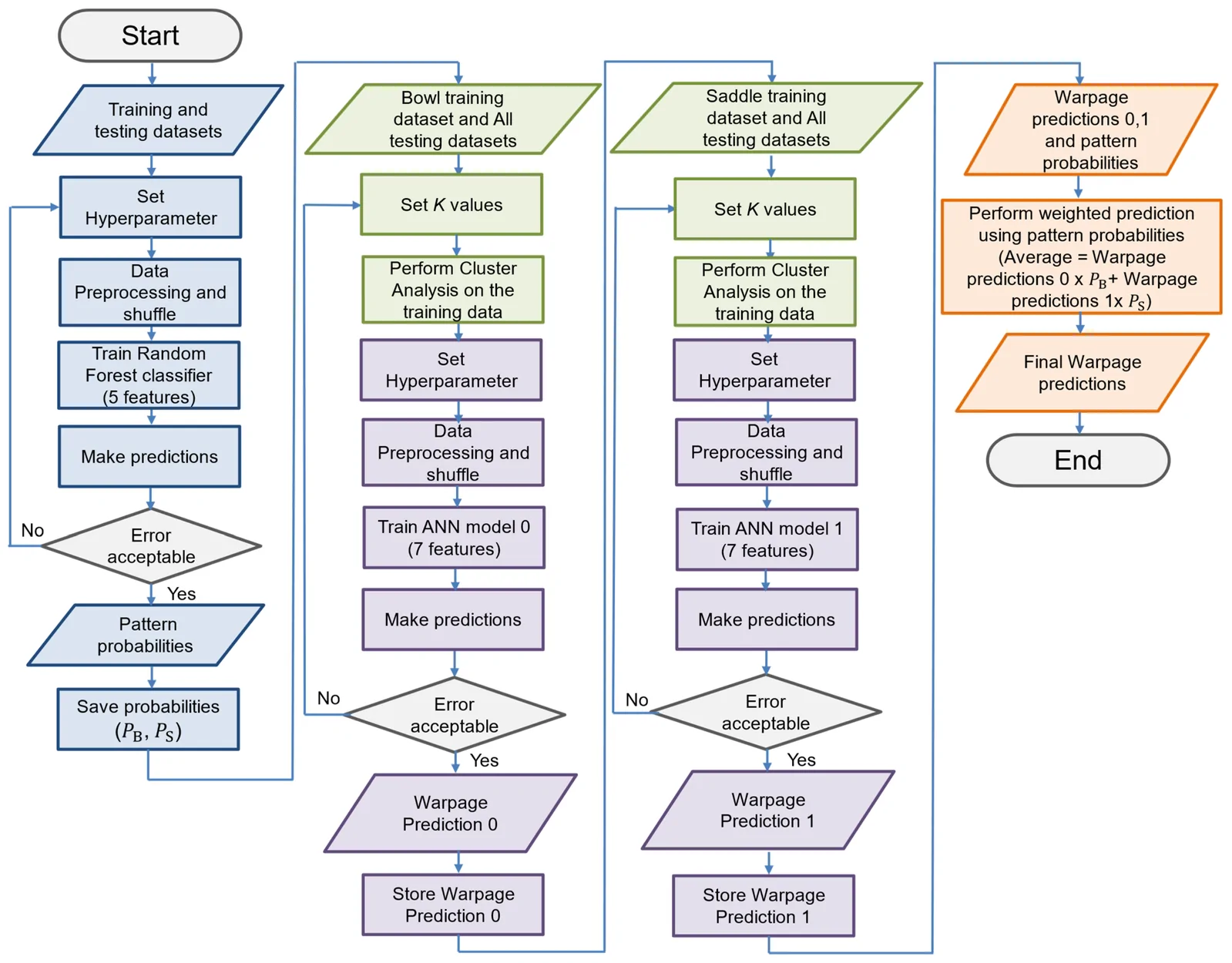
Workflow of the hybrid machine learning method. Blue for pattern probability, green for reducing points, purple for warpage prediction, and orange for weight distribution.

Finally, the outputs of the two ANN models are combined using probability-weighted integration based on the warpage mode probabilities predicted by the Random Forest. Since the predicted probabilities satisfy *P*_B_ + *P*_S_ = 1, the final output can be interpreted as the expected warpage field conditioned on the given geometric parameters. The overall workflow of the proposed hybrid model is illustrated in Figure 15. It consists of three stages: the warpage mode probability estimation, data reduction combined with warpage prediction, and weighted result integration.

The first stage of the proposed workflow is the estimation of the warpage mode probability. A Random Forest model is trained using five geometric features. The hyperparameters used in the Random Forest training are listed in Table 13.

To validate the classification capability of the framework, the Random Forest classifier was evaluated under stratified five-fold cross-validation. The classifier achieves an accuracy of 95.9%, an area under the ROC curve of 0.995, and an out-of-bag accuracy of 98.8%, as detailed in Table 14. Furthermore, it correctly identified the expected mode in every one of the sixteen independent test designs evaluated in this study.

After training and validation, the model is used to predict the probability of possible warpage modes for specific test samples. Test Sample 1 is first input into the trained Random Forest model. As shown in Table 15, the predicted probability for the bowl-shaped pattern is 1, while that for the saddle-shaped pattern is 0, which is consistent with the ground truth. Similarly, Test Sample 2 is then input into the model. The predicted probability for the saddle-shaped pattern is 0.97, also matching the true label. Meanwhile, the bowl-shaped pattern is predicted with a probability of 0.03.

Compared to directly outputting a single class label, providing classification probabilities preserves the model’s confidence in each class, enabling more nuanced and informative decision-making in the subsequent prediction stage. Upon completing the warpage mode probability prediction, the next steps involve the data reduction and warpage prediction stage, followed by the weighted integration stage.

Since different values of *K* can affect the clustering quality and data representation, comparing multiple *K* values provides a more effective reflection of the underlying data characteristics. As noted by Pham et al. [38], when the ratio of *K* to *N* (*K*/*N*) is too small, the clustering structure may be insufficient; conversely, a large *K*/*N* ratio may undermine the purpose of data reduction. In this study, this principle is applied to the spatial warpage field, where *N* denotes the number of spatial data points per sample (1089 points from the 33 × 33 full array).

Therefore, the *K*/*N* ratio is set between 3.33% and 20%, corresponding to *K* values of approximately 30–200. For each fixed *K* value, a grid search is conducted to determine the optimal hyperparameters for the corresponding ANN, enabling a comprehensive comparison of the prediction performance across different *K* values.

**Figure 16:**
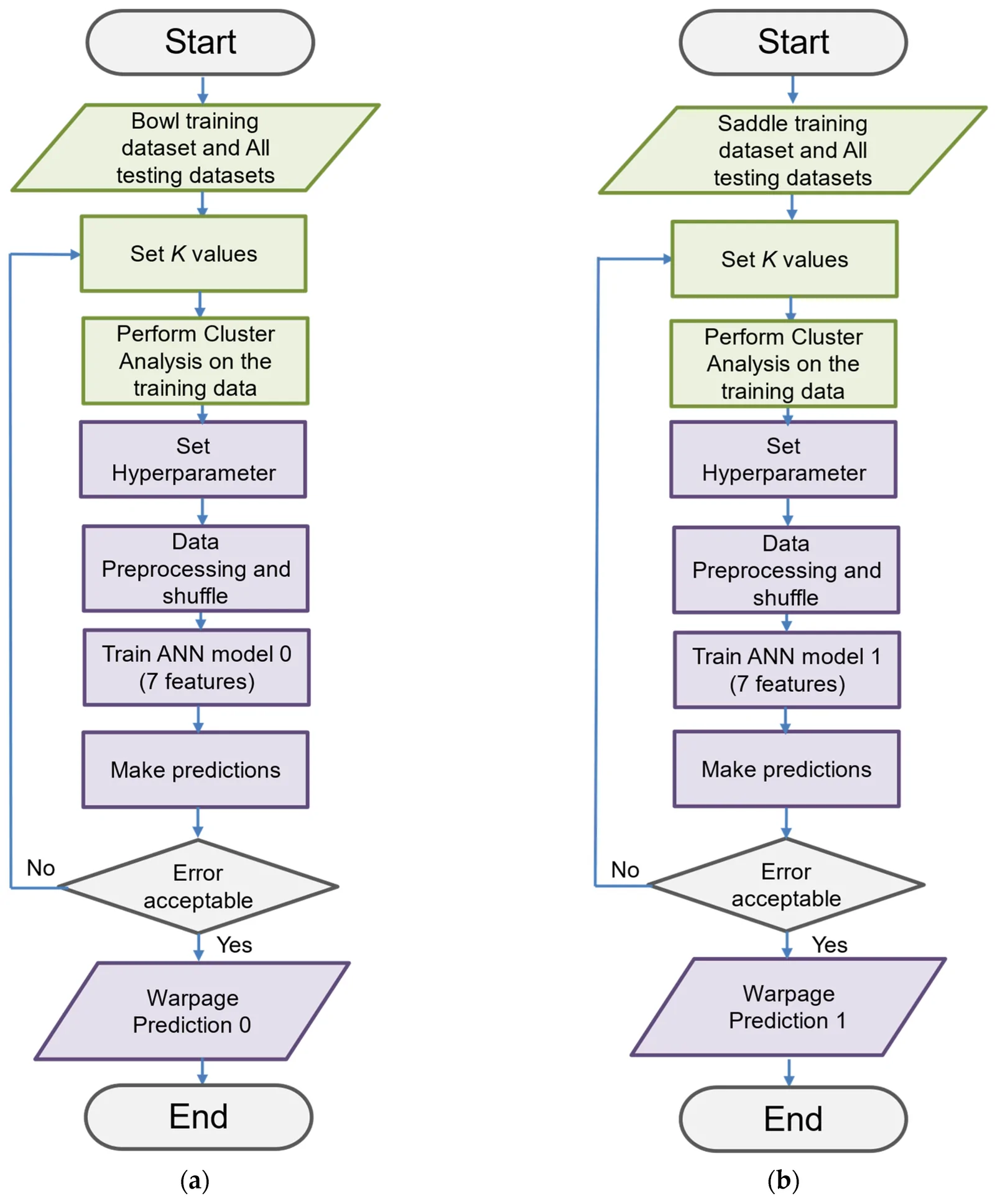
Cluster analysis combined with the two mode-specific networks: (**a**) ANN model 0; and (**b**) ANN model 1. Colors and shapes follow the convention of Figure 15.

The model architecture consists of two distinct neural networks: ANN model 0 and ANN model 1. ANN model 0 is trained using samples with bowl-shaped warpage, while ANN model 1 is trained using samples with saddle-shaped warpage. Accordingly, the impact of *K* is analyzed and compared independently for each model. The workflow combining clustering analysis with neural network prediction is illustrated in Figure 16.

ANN model 0 was initially evaluated at K=30, 40, and 50, with the analysis subsequently extended to K=100 and higher; the complete results are summarized in Table 16. The mean testing error stabilized below 6% for K≥40, while the maximum testing error stabilized for K≥50, with only marginal changes in the prediction performance at larger K values. These results indicate that the predictive accuracy of ANN model 0 converges as K exceeds 50. Accordingly, K=50 was selected as the minimum viable number of clusters for ANN model 0.

ANN model 1 was evaluated over the same range, first at *K* = 30, 40, and 50, and then up to *K* = 100 and beyond; the complete sweep is summarized in Table 17. The mean testing error approaches 3% once *K* exceeds 50, but both the mean and the maximum errors continue to fluctuate until *K* = 70, beyond which they remain essentially unchanged. The prediction accuracy of ANN model 1 therefore stabilizes above *K* = 70, which is taken as the minimum stable *K* for this model. Selecting the smallest *K* at which the accuracy has stabilized avoids unnecessary computation without sacrificing the prediction quality.

The results indicate that, once *K* exceeds a threshold, the prediction accuracy of K-means combined with the ANN stabilizes. Above that threshold, the remaining variation is not systematic. Repeating this evaluation at every *K* in the grid search gives a mean test error of 5.28 ± 0.39% for ANN model 0 and 3.74 ± 0.19% for ANN model 1 above the minimum stable *K*, as shown in Table 18. This confirms the reproducibility of the selected operating point, and that further increases in *K* yield no systematic improvement in accuracy. This phenomenon reflects the diminishing-return regime [43,44,45], which motivates selecting the smallest stable *K*. The point at which the accuracy first stabilizes is therefore adopted as the minimum stable *K*, which maximizes the data reduction while keeping the prediction accuracy within an acceptable range and balancing performance against computational efficiency.

For each model, the selected *K* value corresponds to its minimum viable value. The clustering hyperparameters used are listed in Table 19. For ANN model 0 and ANN model 1, the minimum viable *K* values are 50 and 70, respectively. The optimal hyperparameter settings for each ANN model, obtained through a grid search, are presented in Table 20 and Table 21.

**Figure 17:**
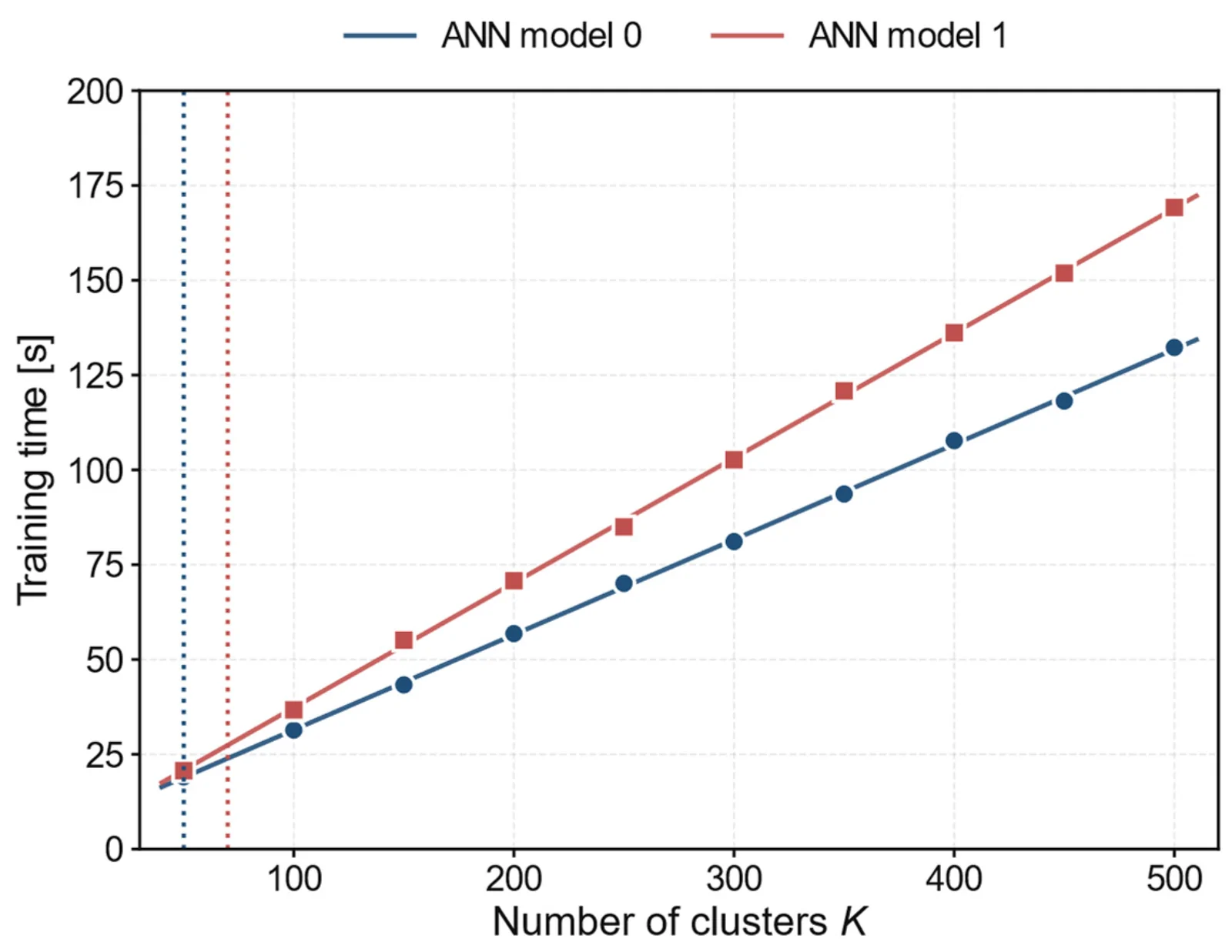
Measured training time of the two mode-specific networks against the number of clusters *K*. The vertical dashed lines represent *K* = 50 (blue) and *K* = 70 (red).

By employing this data selection strategy, the training time was significantly reduced from 443 s required by the original single neural network to approximately 42 s in total (19 s for ANN model 0 and 23 s for ANN model 1). The additional computational cost of Random Forest classification and K-means clustering is relatively minor compared to ANN training, achieving an overall reduction of about 90% in training cost. The training time scales linearly with the number of clusters *K* over the full range tested, as summarized in Table 22 and illustrated in Figure 17. At the selected *K*, the training set falls from 264,627 to 15,190 pointwise entries, a 94.3% reduction, consistent with the 90.4% reduction in network training time.

Next, all test samples are individually input into the two artificial neural network models within the ensemble learning framework. The prediction results from the two models are integrated using a weighted calculation, as defined in Equation (1).

The prediction from ANN model 0, trained on bowl-shaped warpage samples, is denoted as *W*_0_, while the prediction from ANN model 1, trained on saddle-shaped warpage samples, is denoted as *W*_1_. The corresponding Random Forest probabilities are *P*_B_ and *P*_S_, respectively. The final warpage prediction W is obtained as follows:

*W* = *P*_B_ × *W*_0_ + *P*_S_ × *W*_1_, (1)

where *P*_B_ + *P*_S_ = 1.

The results for Test Sample 1 are presented in Table 23. ANN model 0 effectively predicts the warpage due to its training on bowl-shaped warpage data, while ANN model 1, trained exclusively on saddle-shaped warpage, exhibits larger prediction deviations.

**Figure 18:**
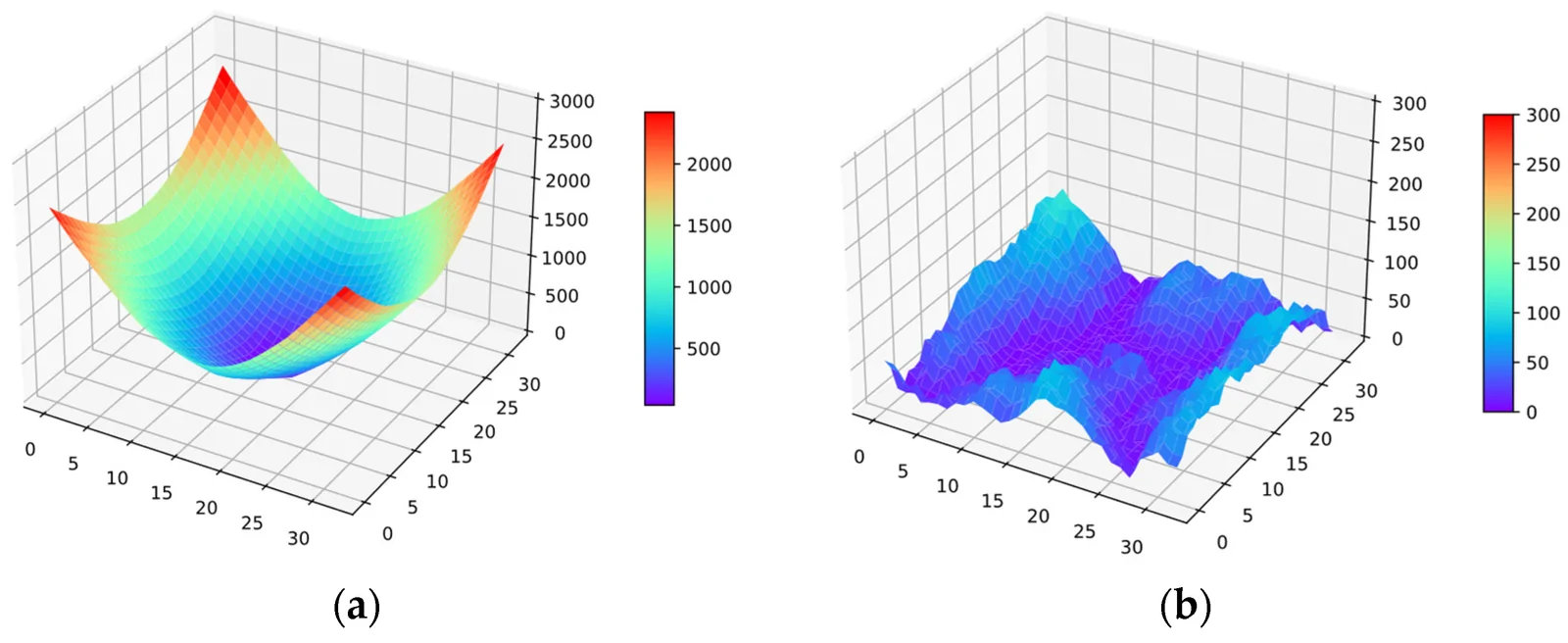
Test Sample 1 predicted by the proposed framework: (**a**) predicted warpage field; and (**b**) absolute error surface. Axes in mm, warpage and error in µm.

By applying ensemble learning, the predictions from both models are weighted according to their respective warpage mode probabilities and then summed. Since the Random Forest predicted *P*_B_ = 1 and *P*_S_ = 0 for Test Sample 1, the ensemble result is mathematically equivalent to the prediction of ANN model 0 alone. The resulting integrated prediction demonstrates an improved accuracy compared to the single ANN baseline, with the average testing error reduced from 6.45% to 4.77%, and the maximum testing error reduced from 10.69% to 5.76%. The predicted warpage distribution and corresponding surface error map are shown in Figure 18.

**Figure 19:**
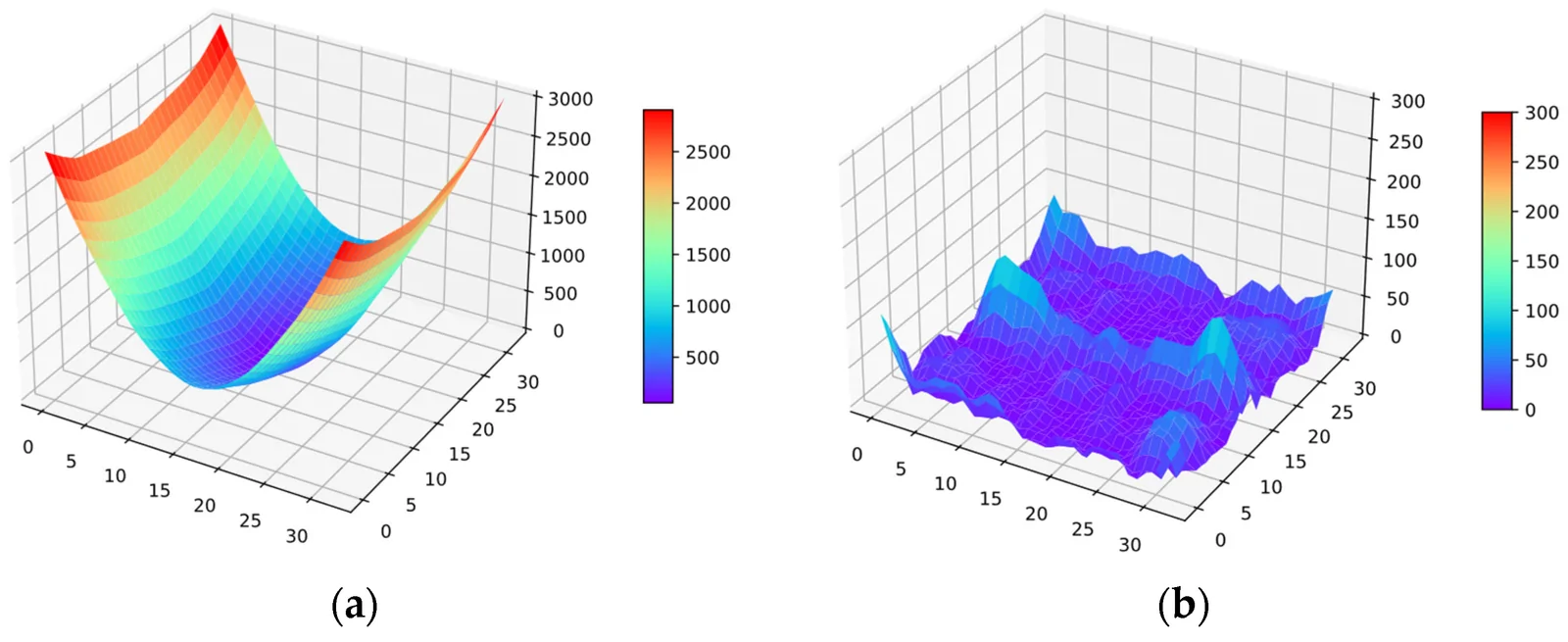
Test Sample 2 predicted by the proposed framework: (**a**) predicted warpage field; and (**b**) absolute error surface. Axes in mm, warpage and error in µm.

The prediction results for Test Sample 2 are also presented in Table 23. Since ANN model 0 was trained solely on bowl-shaped warpage data, its prediction deviated significantly from the actual values. In contrast, ANN model 1 accurately captured the sample’s warpage trend. After weighted integration, the overall prediction performance improved compared to using a single ANN model. The average testing error was reduced to 3.49%, and the maximum testing error was improved to 3.82%. The warpage prediction and corresponding surface error maps are shown in Figure 19.

**Figure 20:**
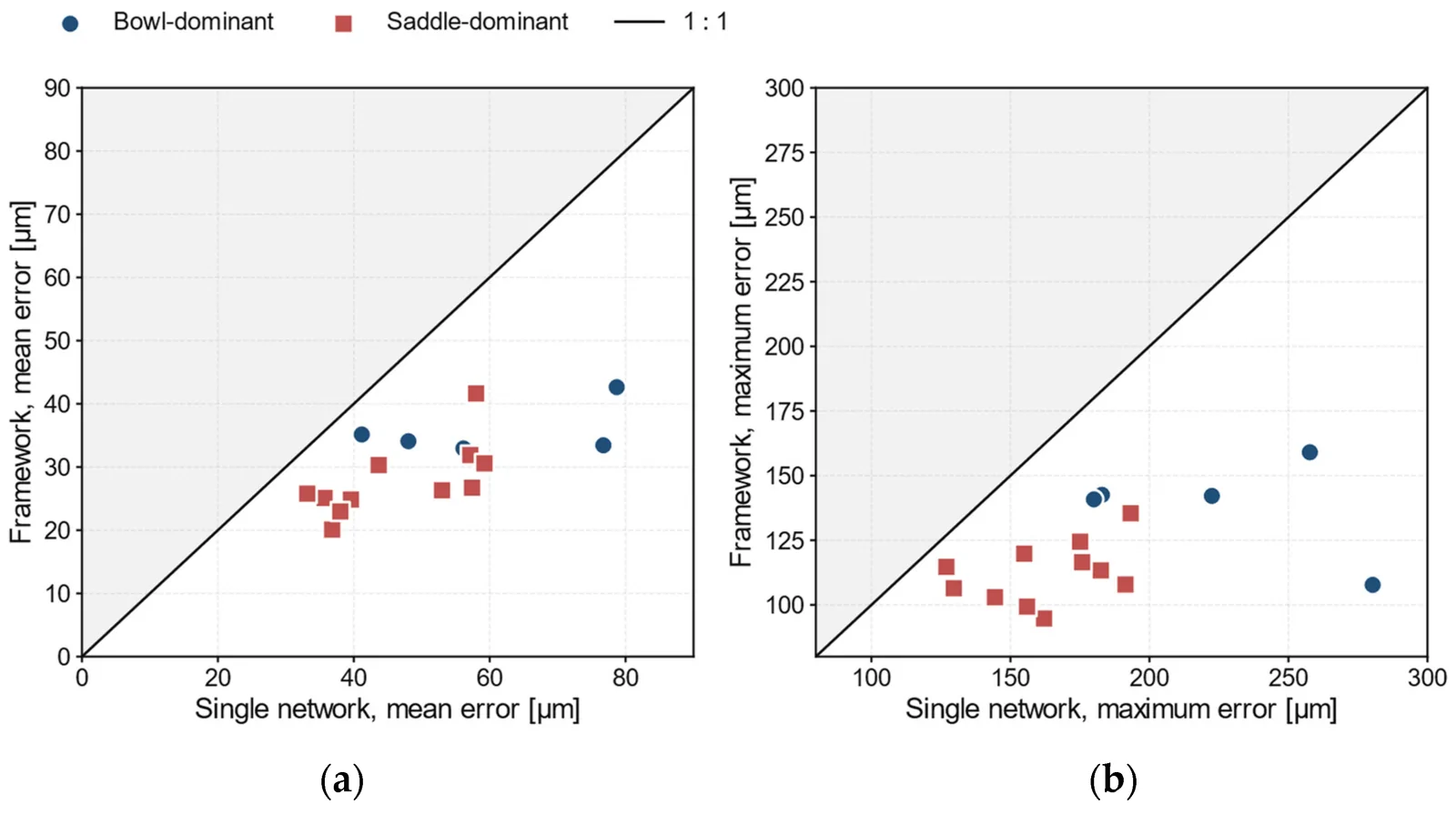
Per-design comparison of the ungated baseline and the proposed framework over the sixteen held-out test designs: (**a**) mean absolute error; and (**b**) maximum absolute error.

To further evaluate the proposed framework across the design space, 16 held-out designs, including five bowl-dominant and eleven saddle-dominant cases, not represented in the training set, were analyzed. Table 24 presents the classifier probability of the saddle-shaped mode and the corresponding prediction errors of both the ungated baseline and the proposed framework, while Table 25 summarizes the overall performance. As shown in Figure 20, the proposed framework achieved lower mean and maximum absolute errors than the baseline for all 16 designs, yielding average reductions of 38.5% and 31.9%, respectively. Moreover, applying a network trained for the opposite warpage mode increased the mean absolute error by a factor of 5.9, from 30.71±5.24 µm to 181.17±64.49 µm, demonstrating the effectiveness of mode-specific network specialization.

**Figure 21:**
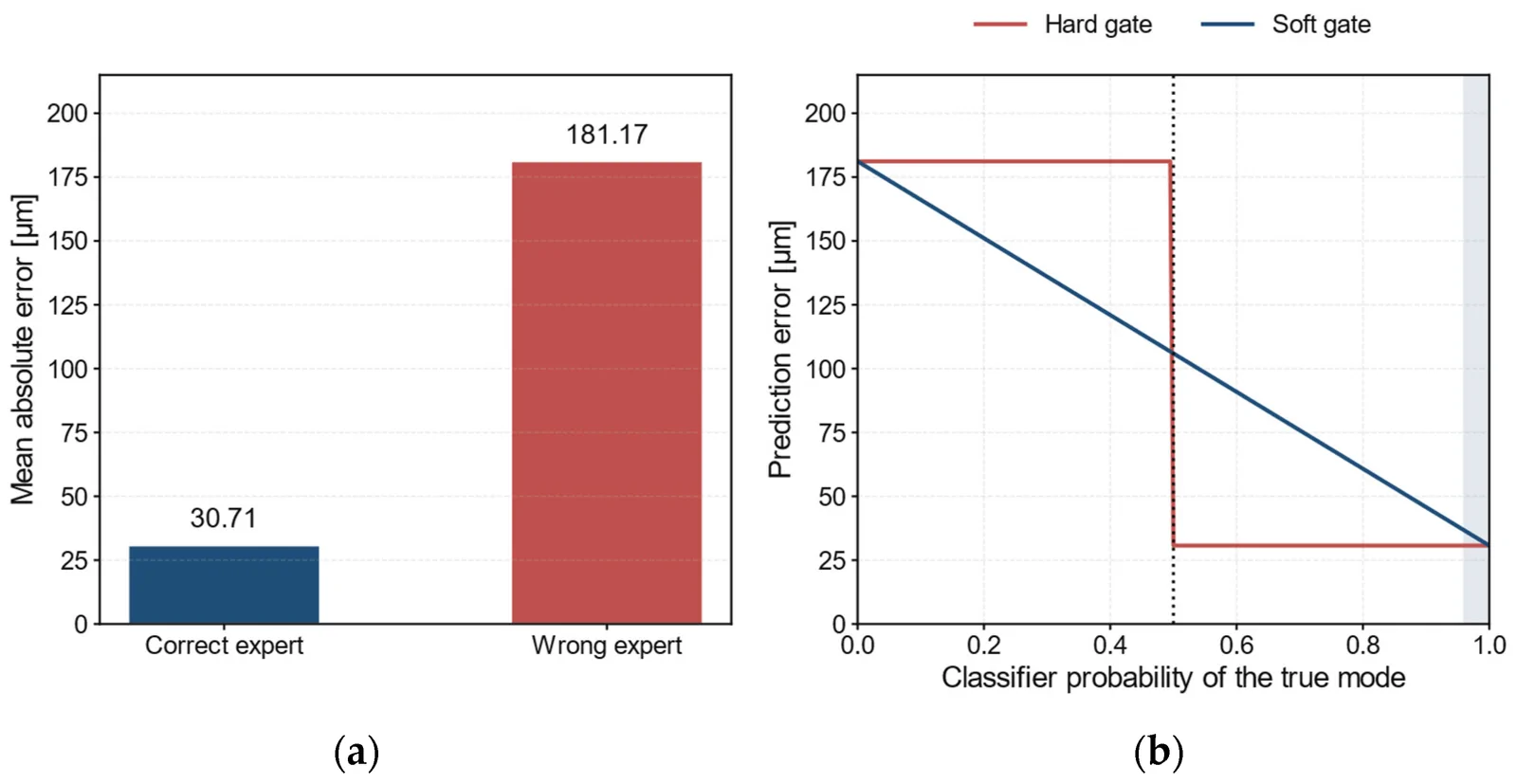
Behavior of the two gating schemes: (**a**) mean absolute error with the correct versus the wrong mode-specific network; and (**b**) prediction error as a function of the classifier probability of the true mode. The vertical dashed line represents the decision threshold where the classifier probability equals 0.5.

An ablation study compared the probability-weighted fusion against a hard arg-max switch over the eleven non-degenerate designs. As detailed in Table 26, the soft gate gave the lower mean error in seven of eleven cases, by 2.82% on average. This benefit is concentrated near the classifier’s decision boundary rather than in the average accuracy for confidently classified designs, as illustrated in Figure 21. Because using the wrong expert is 5.9 times worse, replacing the hard gate with the classifier’s cross-validated accuracy of 95.9% would raise the expected mean error by 20.1%. Thus, retaining the probabilities buys robustness at a negligible cost where the classification is already confident.

## 6. Conclusions

A classifier-gated hybrid machine-learning framework was developed to predict the full warpage field of Fan-Out Panel-Level packages directly from geometric design parameters. The framework employs a Random Forest classifier to estimate the probabilities of the two dominant warpage modes, bowl-shaped and saddle-shaped, while cluster analysis reduces the spatial training data within each mode to a set of representative points. Two mode-specific artificial neural networks are then trained on the reduced datasets and combined through probability-weighted fusion to generate the complete warpage-field prediction. This mode-aware architecture enables specialization for distinct deformation patterns while substantially reducing the training data requirements and computational cost.

The evaluation on 16 held-out designs spanning the design space showed that the proposed framework consistently outperformed an equivalent ungated single-network model, reducing the mean and maximum absolute prediction errors by 38.5% and 31.9%, respectively. The largest improvements occurred at the panel edges and corners, where the warpage is most severe and strongly influenced by the deformation mode. Moreover, applying a network trained for the incorrect warpage mode increased the mean absolute error by a factor of 5.9, confirming the importance of mode-specific specialization. By reducing the effective training dataset by more than 94%, the representative-point strategy also lowered the network training cost by 90.4% without compromising the prediction accuracy, making the framework substantially more efficient than a conventional single-network approach.

The trained framework provides a practical tool for the rapid warpage prediction of new FO-PLP layouts. Once trained, it predicts the full warpage field directly from the geometric design parameters, eliminating the need for repeated finite element simulations and enabling efficient design verification and optimization. Although the current AI model is limited to a fixed material set, it can be readily retrained using the same methodology when the material properties change. Future work could focus on incorporating material systems, including alternative encapsulation materials, to improve the model generalizability across a broader FO-PLP design space.

## Figures and Tables

**Table 1:** Material parameters [22].

Material	CTE [ppm/°C]	Young’s Modulus [GPa]	Poisson’s Ratio	Density [g/cm^3^]	Stress-Free Temperature [°C]
Si	2.8	131	0.28	2.329	25
EMC	7.79–35.9	27.35	0.35	1.8	175
Glass carrier	4.5	73.6	0.23	2.3	25

**Table 2:** Variation of the CTE of EMC with temperature [22].

Temperature [K]	CTE [ppm/°C]
298	7.79
373	10.92

**Table 3:** Constitutive parameters of the rheological model.

Constant	Symbol	Value	Unit
Maxwell dashpot damping coefficient	*η*_m_	20,506.158	N·s/mm
Kelvin–Voigt damping coefficient	*η*_k_	1016.199	N·s/mm
Maxwell spring stiffness	*E*_m_	4.435	N/mm
Kelvin–Voigt spring stiffness	*E*_k_	12.940	N/mm

**Table 4:** Parameter of Prony series.

Creep Modulus	Value
*τ*_1_	6229.821
*τ*_2_	58.303
*G*_m_	0.74
*G*_k_	0.26

**Table 5:** Summary of the finite element model.

Item	Setting
Software/analysis type	ANSYS^®^; large-deflection static analysis, full Newton–Raphson
Panel dimensions	320 × 320 mm; quarter geometry generated and mirrored about both axes
Element type (EMC, die, glass carrier)	SOLID185 (enhanced-strain formulation)
Element type (extra-molding thin layer)	SHELL181 with defined shell section (EMC, 3 integration points)
Element type (vacuum chuck)	COMBIN14 spring elements (massless)
In-plane element size	1.5 mm
Through-thickness discretization	1 element for the die layer, 1 element for the carrier
Number of equations (DOF)	629,136 for the representative design
Approximate element/node count	≈1.0 × 10^5^ SOLID185, 5.2 × 10^4^ SHELL181; ≈1.6 × 10^5^ nodes
Equation solver	Sparse direct solver
Time stepping	Automatic, maximum 100 substeps per load step
Convergence criterion	Displacement-based, tolerance 5%
Solution size	182 load steps; 3288 equilibrium iterations
Process modeling	Element birth and death
Constraint	All degrees of freedom fixed at the panel center node
Body load	Gravity throughout; sign reversed when the panel is inverted before debonding
Thermal history	Molding 175 °C for 1 h, cooling to 25 °C, post-mold cure 175 °C for 4 h, cooling to 25 °C, debonding at 185 °C
Vacuum release	Progressive from the panel edges inward, applied in discrete sub-steps

**Table 6:** Process modeling technology.

Step	Process Description
1	Deactivate the EMC and spring elements. Set the initial temperature of the chip and glass carrier to 25 °C.
2	Heat the active model to the molding temperature of 175 °C.
3	Reactivate the EMC elements at 175 °C to simulate the encapsulation process.
4	Cool the entire model down to 25 °C.
5	Reheat the model to the post-mold cure (PMC) temperature of 175 °C.
6	Cool the model down to 25 °C again.
7	Activate the spring elements (for vacuum simulation), invert the model, and heat it to the debonding temperature of 185 °C.
8	Gradually deactivate the carrier elements from the edges toward the center, then cool the model to 25 °C.
9	Progressively reduce the spring stiffness and deactivate the springs to simulate the final release.

**Table 7:** Five feature sizes.

No.	Feature Name	Symbol
1	Chip thickness	CT
2	Chip size	CS
3	Gap between chips	GS
4	Extra molding thickness	ET
5	Glass carrier thickness	CRT

**Table 8:** Training dataset.

Feature Name	Size [mm]
Chip size (CS)	6, 7, 8
Gap between chips (GS)	1, 1.4, 2
Chip thickness (CT)	0.35, 0.4, 0.45
Glass carrier thickness (CRT)	1.1, 1.3, 1.5
Extra molding thickness (ET)	0.028, 0.042, 0.056
Total data	243
Total data point	243 × 33 × 33 = 264,627

**Table 9:** Testing dataset.

Feature Name	Test Sample 1 [mm]	Test Sample 2 [mm]
Chip size (CS)	6.5	6.5
Gap between chips (GS)	1.25	1.9
Chip thickness (CT)	0.375	0.36
Glass carrier thickness (CRT)	1.2	1.2
Extra molding thickness (ET)	0.035	0.035
Total data	1	1
Total data point	1 × 33 × 33 = 1089	1 × 33 × 33 = 1089

**Table 10:** Grid search range of ANN hyperparameters.

ANN Model	Setting
Preprocessor	StandardScaler
Random state	Fixed
Hidden layers	4
Neuron	100, 150, 200
Activation function	ReLU
Loss function	MSE
Solver	Adam
Learning rate	0.001, 0.0001
Batch size	150, 300, 450
Epoch	100, 200, 300

**Table 11:** Single ANN hyperparameters.

ANN Model	Setting
Preprocessor	StandardScaler
Random state	Fixed
Hidden layers	4
Neuron	100
Activation function	ReLU
Loss function	MSE
Solver	Adam
Learning rate	0.0001
Batch size	300
Epoch	300

**Table 12:** Prediction performance of the single ANN model on Test Sample 1 and Test Sample 2.

Metric	Training	Test Sample 1	Test Sample 2
Mean absolute error [µm]	7.86	65.04	35.20
Mean absolute error [%]	1.34	6.45	5.08
Maximum absolute error [µm]	59.65	265.07	176.48
Maximum absolute error [%]	2.65	10.69	5.96
Reference value at maximum error [µm]	–	2480	2960
Training time [s]	443.32	–	–

**Table 13:** Hyperparameters of Random Forest.

Random Forest	Setting
Random state	Fixed
n_estimators	100
max_depth	None

**Table 14:** Cross-validated classification performance of the Random Forest over the 243 labeled designs.

Metric	Bowl-Shaped	Saddle-Shaped	Overall
Support [designs]	91	152	243
Precision	0.966	0.955	0.959
Recall	0.923	0.980	0.959
F1-score	0.944	0.968	0.959
Confusion matrix	84/7	3/149	–
ROC-AUC	–	–	0.995
Out-of-bag accuracy	–	–	0.988

**Table 15:** Predicted warpage-mode probabilities for the two representative test designs.

Warpage Mode	Test Sample 1	Test Sample 2
Bowl-shaped, *P*_B_	1.00	0.03
Saddle-shaped, *P*_S_	0.00	0.97

**Table 16:** Performance of ANN model 0 with varying *K* values (*K* = 30 to 100, 150, and 200).

*K*	Mean Test Error [µm]	Mean Test Error [%]	Maximum Test Error [µm]	Reference Value at Maximum Error [µm]	Maximum Test Error [%]
30	39.34	6.35	135.74	1520	8.93
40	35.98	5.79	125.39	1680	7.46
50	35.23	4.77	112.96	1960	5.76
60	42.78	5.66	127.36	2050	6.21
70	45.66	5.83	144.79	2480	5.84
80	44.14	5.52	133.07	2480	5.37
90	36.67	5.29	134.16	2480	5.41
100	38.72	4.87	121.85	2050	5.94
150	39.82	4.94	148.23	2480	5.98
200	41.05	5.35	135.30	2250	6.01

**Table 17:** Performance of ANN model 1 with varying *K* values (*K* = 30 to 100, 150, and 200).

*K*	Mean Test Error [µm]	Mean Test Error [%]	Maximum Test Error [µm]	Reference Value at Maximum Error [µm]	Maximum Test Error [%]
30	30.50	5.84	161.99	1680	9.64
40	32.43	6.21	159.24	1680	9.48
50	24.16	4.46	113.57	1680	6.76
60	29.67	4.16	168.08	2960	5.68
70	22.50	3.49	138.62	2960	4.68
80	21.80	3.83	116.71	2960	3.94
90	26.74	3.98	141.37	2960	4.78
100	22.75	3.91	119.34	2960	4.03
150	23.45	3.58	94.97	2960	3.21
200	22.84	3.67	108.23	2960	3.66

**Table 18:** Dispersion of the test error over independently optimized configurations.

Model	Region	n	Mean Error [%]	Maximum Error [%]	Mean Error [µm]	Maximum Error [µm]
ANN model 0	*K* < 50	2	6.07 ± 0.40	8.20 ± 1.04	37.66 ± 2.38	130.56 ± 7.32
ANN model 0	*K* ≥ 50	8	5.28 ± 0.39	5.82 ± 0.29	40.51 ± 3.61	132.22 ± 11.53
ANN model 1	*K* < 70	4	5.17 ± 1.01	7.89 ± 1.98	29.19 ± 3.55	150.72 ± 25.04
ANN model 1	*K* ≥ 70	6	3.74 ± 0.19	4.05 ± 0.60	23.35 ± 1.75	119.87 ± 17.77

**Table 19:** Hyperparameter of *K*-means.

*K*-Means	Setting
init	*K*-means++
n_cluster	50 (ANN model 0) 70 (ANN model 1)
Max_iter	Default (300)
tol	Default (1 × 10^−4^)

**Table 20:** Optimal hyperparameters of ANN model 0 at *K* = 50.

ANN Model 0	Setting
Preprocessor	StandardScaler
Random state	Fixed
Hidden layers	4
Neuron	150
Activation function	ReLU
Loss function	MSE
Solver	Adam
Learning rate	0.001
Batch size	150
Epoch	300

**Table 21:** Optimal hyperparameters of ANN model 1 at *K* = 70.

ANN Model 1	Setting
Preprocessor	StandardScaler
Random state	Fixed
Hidden layers	4
Neuron	100
Activation function	ReLU
Loss function	MSE
Solver	Adam
Learning rate	0.001
Batch size	300
Epoch	300

**Table 22:** Training points, iterations, and measured training time versus the number of clusters.

*K*	Model 0 Points	Model 0 Iterations	Model 0 Time [s]	Model 1 Points	Model 1 Iterations	Model 1 Time [s]
50	4550	9300	19.06	7600	10,200	20.69
100	9100	18,300	31.39	15,200	20,400	36.72
150	13,650	27,300	43.31	22,800	30,600	55.12
200	18,200	36,600	56.83	30,400	40,800	70.77
250	22,750	45,600	70.07	38,000	51,000	84.97
300	27,300	54,600	81.09	45,600	61,200	102.66
350	31,850	63,900	93.64	53,200	71,400	120.85
400	36,400	72,900	107.72	60,800	81,600	136.18
450	40,950	81,900	118.16	68,400	91,800	151.89
500	45,500	91,200	132.29	76,000	102,000	169.21

**Table 23:** Performance of the mode-specific networks and of the fused prediction on the two representative test designs.

Quantity	ANN Model 0 (*K* = 50)	ANN Model 1 (*K* = 70)	Fused Prediction
Training mean error [µm/%]	15.69/2.07	8.65/1.21	–
Training max error [µm/%]	115.61/5.56	87.74/4.22	–
Training time [s]	19.43	23.30	42.73
Test Sample 1, mean error [µm/%]	35.23/4.77	158.91/16.68	35.23/4.77
Test Sample 1, maximum error [µm/%]	112.96/5.76	550.25/37.43	112.96/5.76
Test Sample 1, reference at maximum error [µm]	1960	1470	1960
Test Sample 2, mean error [µm/%]	344.99/36.22	22.50/3.49	19.20/3.49
Test Sample 2, maximum error [µm/%]	1109.01/43.32	138.62/4.68	112.99/3.82
Test Sample 2, reference at maximum error [µm]	2560	2960	2960

**Table 24:** Ungated baseline and proposed framework on sixteen held-out designs.

Design	Mode	*P*_S_	Single MAE [µm]	Single Max [µm]	Framework MAE [µm]	Framework Max [µm]
CT0.375CS6.5GS1.25ET0.035CRT1.4	bowl	0.01	56.14	222.45	32.94	142.15
CT0.375CS6.5GS1.25ET0.049CRT1.2	bowl	0.00	76.73	280.29	33.44	107.75
CT0.375CS6.5GS1.25ET0.049CRT1.4	bowl	0.02	78.69	257.70	42.63	159.04
CT0.425CS6.5GS1.25ET0.049CRT1.2	bowl	0.03	41.18	182.92	35.15	142.56
CT0.425CS6.5GS1.25ET0.049CRT1.4	bowl	0.02	48.04	180.02	34.08	140.81
CT0.375CS6.5GS1.75ET0.035CRT1.2	saddle	0.99	39.59	175.06	24.85	124.45
CT0.375CS6.5GS1.75ET0.035CRT1.4	saddle	0.96	36.82	129.62	20.10	106.43
CT0.375CS6.5GS1.75ET0.049CRT1.2	saddle	0.99	35.70	182.53	25.09	113.34
CT0.375CS6.5GS1.75ET0.049CRT1.4	saddle	0.99	33.15	126.97	25.78	114.71
CT0.375CS7.5GS1.25ET0.049CRT1.2	saddle	0.99	58.01	193.24	41.63	135.41
CT0.375CS7.5GS1.25ET0.049CRT1.4	saddle	1.00	43.67	144.41	30.30	102.96
CT0.375CS7.5GS1.75ET0.049CRT1.2	saddle	1.00	52.98	162.08	26.32	94.74
CT0.375CS7.5GS1.75ET0.049CRT1.4	saddle	1.00	57.40	155.97	26.72	99.33
CT0.425CS6.5GS1.75ET0.049CRT1.2	saddle	0.99	38.02	154.95	22.96	119.79
CT0.425CS7.5GS1.75ET0.049CRT1.2	saddle	0.99	57.14	191.37	31.88	107.89
CT0.425CS7.5GS1.75ET0.049CRT1.4	saddle	1.00	59.22	175.79	30.57	116.49

**Table 25:** Aggregate performance over the sixteen designs (mean ± standard deviation).

Group	n	Single MAE [µm]	Single Max [µm]	Framework MAE [µm]	Framework Max [µm]	Mean Error Gain	Maximum Error Gain
All designs	16	50.78 ± 13.82	182.21 ± 41.89	30.28 ± 6.33	120.49 ± 18.50	38.5%	31.9%
Bowl-dominant	5	60.15 ± 16.89	224.68 ± 44.52	35.65 ± 3.99	138.46 ± 18.73	37.5%	36.0%
Saddle-dominant	11	46.52 ± 10.42	162.91 ± 22.98	27.84 ± 5.73	112.32 ± 11.75	39.0%	30.1%

**Table 26:** Probability-weighted fusion compared with hard switching over the eleven non-degenerate designs.

Group	n	Soft Better on Mean Error	Gain in Mean Error	Soft Better on Maximum Error	Gain in Maximum Error
All non-degenerate	11	7 (64%)	+2.82%	6 (55%)	+1.72%
Saddle-dominant	7	7 (100%)	+6.41%	6 (86%)	+3.90%
Bowl-dominant	4	0 (0%)	−3.45%	0 (0%)	−2.08%

## Data Availability

The original contributions presented in this study are included in the article. Further inquiries can be directed to the corresponding author.

## Nomenclature

The following symbols are used in this manuscript:

*E*_m_	Maxwell spring stiffness	[N/mm]
*E*_k_	Kelvin–Voigt spring stiffness	[N/mm]
*η*_m_	Maxwell dashpot damping coefficient	[N·s/mm]
*η*_k_	Kelvin–Voigt damping coefficient	[N·s/mm]
*G*_m_	Prony relative modulus, first term (0.74)	[–]
*G*_k_	Prony relative modulus, second term (0.26)	[–]
*τ*_1_	Prony relaxation time, first term (6229.821)	[s]
*τ*_2_	Prony relaxation time, second term (58.303)	[s]
*P*_B_	Predicted probability of the bowl-shaped mode	[–]
*P*_S_	Predicted probability of the saddle-shaped mode	[–]
*W*	Fused predicted warpage field	[µm]
*W*_0_	Warpage predicted by ANN model 0	[µm]
*W*_1_	Warpage predicted by ANN model 1	[µm]
*N*	Number of spatial points per design (1089)	[–]
*K*	Number of clusters in K-means	[–]
CT	Chip thickness	[mm]
CS	Chip size	[mm]
GS	Gap between dies	[mm]
ET	Extra molding thickness	[mm]
CRT	Carrier thickness	[mm]

## Abbreviations

The following abbreviations are used in this manuscript:

ANN	Artificial neural network
CTE	Coefficient of thermal expansion
EMC	Epoxy molding compound
FEM/FEA	Finite element method/analysis
FO-PLP	Fan-Out Panel-Level packaging
MAE	Mean absolute error
MAPE	Mean absolute percentage error
MSE	Mean squared error
PMC	Post-mold cure
RDL	Redistribution layer
ReLU	Rectified linear unit
RF	Random Forest
WLP	Wafer-level packaging
